# Genetically predicted sex hormone binding globulin and ischemic heart disease in men and women: a univariable and multivariable Mendelian randomization study

**DOI:** 10.1038/s41598-021-02510-w

**Published:** 2021-11-30

**Authors:** Jie V. Zhao, C. Mary Schooling

**Affiliations:** 1 School of Public Health, Li Ka Shing, Faculty of Medicine, The University of Hong Kong, 1/F, Patrick Manson Building, 7 Sassoon Road, Hong Kong, SAR China; 2 School of Public Health and Health Policy, City University of New York, New York, NY USA

**Keywords:** Cardiology, Endocrinology

## Abstract

Men are more vulnerable to ischemic heart disease (IHD) than women, possibly due to testosterone. Correspondingly, sex hormone binding globulin (SHBG) which lowers circulating testosterone might protect men against IHD. SHBG may also affect IHD independent of testosterone, which has not previously been examined. To assess the sex-specific role of SHBG in IHD, in univariable Mendelian randomization (MR), we used sex-specific, genome-wide significant genetic variants to predict SHBG, and examined their association with IHD in the UK Biobank. We also replicated using genetic instruments from Japanese men and applied to Biobank Japan. To assess the role of SHGB independent of testosterone in men, we used multivariable MR controlling for testosterone. Genetically predicted SHBG was associated with lower IHD risk in men [odds ratio (OR) 0.78 per standard deviation, 95% confidence interval (CI) 0.70 to 0.87], and the association was less clear in women. The estimates were similar in Japanese. The inverse association remained after controlling for testosterone in men (OR 0.79, 95% CI 0.71 to 0.88). SHBG might lower the risk of IHD in men, with a role independent of testosterone. Exploring intervention strategies that increase SHBG is important for targeting IHD treatments.

## Introduction

Ischemic heart disease (IHD), the leading cause of death worldwide^1^, poses a major burden to global health. For decades it has been observed that at every age, men are more likely to die from IHD than women^2,3^, which contributes to shorter life expectancy in men than women. The importance of considering sex and gender in clinical practice, precision medicine and research is increasingly recognized^4^. Reasons underlying this sex disparity have not been fully elucidated, and are not fully explained by well-established risk factors, such as smoking, physical activity, blood pressure and lipid profile^5,6^. The main sex hormone in women, estrogen, has been thoroughly investigated as a potential causal factor for IHD^7^, however, large randomized controlled trials (RCT) in men, the Coronary Drug Project^8^, and in women, the Women's Health Initiative, did not support a role of estrogen in IHD^9^. Alternatively, accumulating evidence from meta-analysis of RCTs and Mendelian randomization (MR) studies suggest that testosterone, the main sex hormone in men, may partly explain the sex disparity^10–13^. Sex hormone binding globulin (SHBG), which binds to testosterone, lowers bioavailable testosterone^14^, so could also play a role in IHD. Notably, SHBG might be more than a modulator of sex hormones, but may also exert an effect independent of sex hormones^15^.

Observationally, men with lower serum SHBG have lower risk of cardiovascular events, and the association is independent of testosterone^16,17^, although the association is not evident in all observational studies^18^. In contrast, in women, higher serum SHBG is associated with lower risk of cardiovascular events^19^ or has no association with cardiovascular events^20^. SHBG increases with age, and might be affected by socioeconomic position and lifestyle^21,22^, so in conventional observational studies, it is difficult to determine whether the observed association is causal, or a reflection of residual confounding, as in the controversial evidence from observational studies versus RCTs on the role of testosterone and estrogen^10,23^. To our knowledge, no large RCT examining the effect of SHBG on IHD overall or by sex exists.

In this situation where experimental evidence is unavailable, a Mendelian Randomization (MR) study design has been increasingly used in recent years as an alternative way to provide unconfounded estimates^24^. Specifically, MR uses genetic variants which result in life-long differences in exposure (here SHBG) as an instrument, to examine whether people with genetically predicted higher exposure have higher or lower risk of disease (here IHD). As the genetic variants are determined at conception, MR minimizes confounding by socioeconomic position or other confounders, and obtains estimates without any potentially harmful interventions^25^. Previous MR studies have consistently shown a protective association of genetically predicted SHBG with type 2 diabetes^26–28^, cancer^29^ and chronic kidney disease^30^ and IHD^31,32^. The sex-specific role of SHBG independent of testosterone has not been examined in MR. A recent sex-specific genome-wide association study (GWAS) of SHBG and testosterone in by far the largest cohort in Europeans, the UK Biobank^33^, provides an opportunity to assess the sex-specific associations of SHBG with IHD, as well as the role of SHBG independent of testosterone.

Here, we used univariable MR to examine the role of genetically predicted SHBG in IHD in men and women separately, by applying published, sex-specific, genome-wide significant genetic variants predicting SHBG^14^ to sex-specific genetic associations with IHD in men and women from the UK Biobank^33^. We repeated the analysis using another set of genetic variants for SHBG from the *SHBG* gene identified in another previous GWAS^34^. We also similarly examined the role of SHBG in IHD in Japanese men, using genome-wide significant genetic variants for SHBG in Japanese men^35^ and applied to Biobank Japan^36^. Finally, to examine the role of SHBG independent of testosterone, we used multivariable MR to control for bioavailable testosterone.

## Methods

### Study design

We used univariable MR to assess the total effect of SHBG on IHD in white British from the UK Biobank sex-specifically, using two set of genetic variants in Europeans, i.e., all sex-specific, genome-wide significant, independent (r^2^ < 0.05) genetic variants predicting SHBG^33^, and genome-wide significant genetic variants predicting SHBG in the *SHBG* gene^34^. We also assessed the role of SHBG in Japanese men, using genetic variants in *SHBG* gene predicting SHBG (rs2075230 and rs727428) validated in Japanese men^35^, applied to Biobank Japan^36^.

The MR estimates were based on Wald estimates^37,38^, i.e., genetic associations with IHD divided by genetic associations with SHBG. We used multivariable MR to estimate the effect of SHBG after controlling for bioavailable testosterone^39^, by additionally including genetic predictors for bioavailable testosterone (shown in Supplementary Fig. S1).Details of the data sources were shown in Supplementary Table S1.

### Genetic predictors for SHBG in univariable MR

MR depends on three assumptions, i.e., the genetic variants (1) are strongly associated with the exposure, (2) are not subject to confounding, and (3) only influence the outcome via affecting the exposure^40^ without selection bias^41^. As previously^30^, to satisfy the first assumption, for Europeans we used genetic variants predicting SHBG (natural log transformed) in men and women^14^, from the largest sex-specific GWAS conducted in the UK Biobank (178,782 white British men and 230,454 white British women). The GWAS was also replicated in three independent studies (CHARGE Consortium, Twins UK and EPIC-Norfolk)^14^. The GWAS provided 357 genome-wide significant (*p* < 5 × 10^–8^), uncorrelated (r^2^ < 0.05) genetic variants for SHBG in men and 359 uncorrelated SNPs for SHBG in women, with minor allele frequency > 0.1%, imputation quality score > 0.5^14^, as shown in Supplementary Tables S2 and S3. Genetic correlation between men and women for SHBG was 0.83^14^. To satisfy the second assumption, we checked their associations with potential confounders, including Townsend index, smoking, alcohol drinking, and physical activity, using the UK Biobank summary statistics, to identify whether any genetic variant was relevant to these potential confounders at genome-wide significance (*p* < 5 × 10^–8^) (Supplementary Tables S4 and S5). To satisfy the third assumption, we checked whether any genetic variant was directly associated with IHD, or with factors causing IHD but not on the pathway from SHBG to IHD. Low density lipoprotein (LDL)-cholesterol, body mass index (BMI), type 2 diabetes, and blood pressure, and lipoprotein (a) are well-established casual factors for IHD^42^. Previous studies suggest that BMI, type 2 diabetes and blood pressure might be affected by SHBG^14,30^, whilst LDL-cholesterol and lipoprotein (a) might not (Supplementary Table S6)^28^. As such, genetic variants associated with LDL-cholesterol or lipoprotein (a) may affect IHD other than via SHBG. To minimize such pleiotropy, we checked and excluded SNP(s) related to LDL-cholesterol or lipoprotein (a) at genome-wide significance. BMI is a downstream factor of SHBG^14^, so it might be on the pathway from SHBG to IHD, whilst it may also affect SHBG^43^. Given that uncertainty, we also identified the genetic variants related to BMI at genome-wide significance, and excluded them in sensitivity analysis. Details of the selected SNPs were shown in Supplementary Tables S2 and S3.

For replication, we used a second set of published genetic variants from another GWAS conducted in 21,791 participants (12,401 men, 9390 women) and validated in 7,046 participants (2,537 men, 4,509 women) in another six studies^34^. As previously^44^, we used the 3 genetic variants in the *SHBG* gene. As the GWAS was not conducted in men and women separately, we obtained sex-specific association of each SNP with SHBG in the UK Biobank.

To examine the role of SHBG in Asians, we used genetic variants predicting SHBG at genome-wide significance replicated in Japanese men, specifically rs2075230 and rs727428 in the *SHBG* gene^35^. The study was conducted in two cohorts of Japanese men, 901 young men (mean age 20.7 years) as the discovery cohort and 786 men (mean age 31.2 years) as the validation cohort, with adjustment for age and BMI. We could not examine the role of SHBG in Japanese women, because genetic variants predicting SHBG in Japanese women are unavailable.

### Genetic associations with IHD in the UK Biobank and Biobank Japan

Genetic associations with IHD in Europeans were obtained from individual-level data in the UK Biobank (under application #42468). The UK Biobank is a large, ongoing, prospective cohort study, with median follow up time of 12.1 years^33^. The UK Biobank recruited 502,713 people (aged 40–69 years, mean age 56.5 years, 45.6% men) from 2006 to 2010 from England, Scotland and Wales, 94% of self-reported European ancestry. As previously^45^, to control for population stratification, we restricted our analysis to participants of white British ancestry, and excluded people with withdrawn consent, inconsistent gender information between questionnaire and genotyping, excess relatedness (more than 10 putative third-degree relatives), abnormal sex chromosomes (such as an extra X chromosome) or poor-quality genotyping (heterozygosity or missing rate > 1.5%).

IHD events included mortality, hospitalization and self-reported IHD (ICD10-code: I20-25, ICD9-code: 410-414, and self-report code: 1074, 1075). Information was obtained from a nurse-led interview at recruitment, ongoing follow-up via record linkage to inpatient admissions and death registration^33^, updated to Dec 2019. Sex-specific logistic regression for each genetic variant with IHD was adjusted for age, 20 principal components and assay array (the UK BiLEVE array and UK Biobank Axiom array).

Genetic associations with IHD in Japanese men were obtained from summary statistics in Biobank Japan, one of the largest East Asian biobanks, with 21,611 IHD cases and 87,736 controls in men and 7,708 cases and 95,398 controls in women^36^. Genetic associations were obtained from a generalized linear mixed model using SAIGE, with 8,712,794 autosomal variants and 207,198 variants in X chromosome for the association with 42 diseases^36^.

### Statistical analysis for MR estimates using univariable MR

After aligning the SNPs on allele letter, given both studies used the same coding, we meta-analyzed the Wald estimates using inverse variance weighting (IVW) with multiplicative random effects. The strength of these genetic instruments was assessed from the F-statistic, calculated as the square of genetic variant-exposure associations divided by the square of its standard error^46^. To examine whether the MR estimates were different in men and women, we calculated the *p* value for the differences in sex-specific estimates using a well-established formula to calculate the z-statistic, and then obtained the two-tailed *p* value^47^.

In sensitivity analysis, we used leave-one-out analysis to assess whether the association was driven by a single SNP. We also used other statistical methods robust to pleiotropy, specifically, a weighted median, MR-Egger, and a mode-based method. The weighted median is robust to invalid instruments and provides consistent estimation even when up to 50% of the weight is from invalid genetic variants^48^. MR-Egger is robust to invalid instruments as long as the pleiotropic does not act via a confounder of exposure on outcome, i.e., the InSIDE (INstrument Strength Independent of Direct Effect) assumption is satisfied^49^. The mode-based method assumes the true causal effect is the value taken by the largest number of genetic variants^50^, so it is robust to outliers^51^. Given the potential bias arising from overlapping samples of genetic association with exposure, and with the outcome^52^, we replicated the analysis where we obtained the sex-specific genetic association with SHBG from a random subset of UK Biobank without IHD in 50,000 men and 50,000 women, as well as with IHD from the rest of the biobank.

For the power calculation we used the approximation that the sample size required for MR is the sample size needed in the conventional observational study divided by R^2^ (the variance in SHBG explained by the genetic variants)^53^. The required sample size for IHD was calculated based on the odds ratio, the ratio of cases to non-cases, and the R^2^^54^.

### Genetic predictors for multivariable MR controlling for testosterone

To assess the effect of SHBG independent of bioavailable testosterone, we used multivariable MR controlling for bioavailable testosterone. Specifically, we included the genetic predictors for SHBG and the genetic predictors for bioavailable testosterone from the UK Biobank GWAS^14^. After combining the genetic predictors for SHBG and bioavailable testosterone, we dropped duplicate genetic variants and checked the remaining for correlations using MR-Base “ld_clump”, and LDlink (https://ldlink.nci.nih.gov/). We dropped correlated genetic variants (r^2^ > 0.05) and those whose correlations were not available. The remaining were used in multivariable MR.

### Statistical analysis for MR estimates using multivariable MR

We obtained multivariable MR estimate using multivariable IVW, an extension of the inverse-variance weighted method for multivariable MR, using multivariable weighted linear regression^39^. To assess instrument strength, we calculated the conditional F-statistic^55^. To assess potential pleiotropy, we estimated the modified Cochran’s Q statistic^55^. The conditional F-statistic and Cochrane’s Q statistic were calculated using the R package “MVMR”, taking into account the covariance of testosterone and SHBG. We also tested directional pleiotropy orientated on SHBG by examining the significance of the multivariable MR Egger intercept^56^, where a non-zero intercept is indicative of directional pleiotropy. Multivariable MR Egger can also provide a corrected estimate when such pleiotropy exists. In sensitivity analysis, we also used the multivariable MR median, an extension of weighted median to MVMR^57^. The analysis was conducted using the R package “MendelianRandomization”.

All statistical analyses were conducted using R version 4.0.1 (R Foundation for Statistical Computing, Vienna, Austria).

### Ethical approval

This research has been conducted using the UK Biobank Resource under Application number 42468. No original data were collected for the MR study. Ethical approval for each of the studies included in the investigation can be found in the original publications (including informed consent from each participant). The UK Biobank has already received the ethical approval from North West Multi-centre Research Ethics Committee (MREC) which covers the UK. It also got the approval from the Patient Information Advisory Group (PIAG) in England and Wales, and from the Community Health Index Advisory Group (CHIAG) in Scotland. The study conforms to the ethical guidelines of the 1975 Declaration of Helsinki. The analysis of other publicly available summary statistics does not require additional ethical approval.

### Ethics approval and consent to participate

This research has been conducted using the UK Biobank Resource under Application number 42468. No original data were collected for the MR study. Ethical approval for each of the studies included in the investigation can be found in the original publications (including informed consent from each participant). The UK Biobank has already received ethical approval from the Research Ethics Committee and participants provided written informed consent. The analysis of other publicly available summary statistics does not require additional ethical approval.

## Results

In univariable MR, after excluding pleiotropic SNPs related to LDL-cholesterol or lipoprotein (a), we used 343 genome-wide significant SNPs for SHBG in men and 330 SNPs for SHBG in women (Supplementary Tables S2 and S3). All SNPs had F-statistic > 10, with average F-statistic 176.8 in men and 120.8 in women, with r^2^ of around 0.04 in men and 0.03 in women. One SNP (rs1260326) was associated with alcohol drinking and also related to LDL-cholesterol, so it was excluded in main analysis (Supplementary Tables S4 and S5). 4 SNPs were related to BMI in men and 2 SNPs were related to BMI in women (Supplementary Tables S2 and S3), which were excluded in sensitivity analysis. We replicated using 3 SNPs (rs12150660, rs1625895 and rs1641537) in *SHBG*. In the UK Biobank we identified 25,409 IHD cases in 179,911 white British men and 12,511 IHD cases in 212,074 white British women (Supplementary Table S1). At an R^2^ of around 0.04 in men and 0.03 in women, the UK Biobank has 0.8 power to detect an odds ratio (OR) of 0.91 for IHD per one standard deviation increase in SHBG in men and an OR of 0.86 for IHD in women^54^.

Using univariable MR, genetically predicted SHBG was associated with lower risk of IHD in men, but had null associations with IHD in women (Table 1). The scatter plot was shown in Supplementary Fig S2. The difference by sex was not significant (*p* value for sex difference 0.32). The findings were consistent using the 3 SNPs in the *SHBG* gene (Table 2). The associations were robust to using different analytic methods (Tables 1, 2), and robust to excluding SNPs related to BMI (Supplementary Table S7). Leave-one-out sensitivity analysis also indicated the results were not driven by a single SNP (Supplementary Fig. S1). The estimates were similar when using non-overlapping samples in the UK Biobank (Supplemental Table S8). Genetically predicted SHBG was similarly associated with IHD in Japanese men (Table 2), but the confidence interval is wider and includes the null, due to the relatively less power in Japanese.

**Table 1:** Sex-specific associations of genetically predicted SHBG with ischemic heart disease in the UK Biobank using univariable MR.

Sex	#SNPs	Methods	OR	95% CI	*p*	MR Egger intercept *p*
Men	343	IVW	0.78	0.70, 0.87	1.4 × 10^–5^	
		WM	0.79	0.70, 0.91	0.001	
		MR Egger	0.81	0.70, 0.95	0.007	0.50
		MBE	0.82	0.72, 0.92	0.001	
Women*	330	WM	0.89	0.74, 1.08	0.25	
		MR Egger	0.90	0.76, 1.08	0.25	0.04
		MBE	0.94	0.80, 1.10	0.45	

IVW, inverse variance weighting; WM, weighted median; MBE, mode-based estimation.

*IVW was not used in women because MR Egger intercept *p* < 0.05, so corrected estimates from MR Egger instead of IVW was used.

**Table 2:** Associations of genetically predicted SHBG with ischemic heart disease in the UK Biobank and Biobank Japan using genetic variants in *SHBG* gene.

Sex	Source	#SNPs	Methods	OR	95% CI	*p*
Men	UK Biobank	3	IVW	0.91	0.85, 0.97	0.002
			WM	0.91	0.85, 0.97	0.004
	Biobank Japan	2	IVW*	0.91	0.83, 1.002	0.056
Women	UK Biobank	3	IVW	0.96	0.85, 1.07	0.44
			WM	0.96	0.85, 1.08	0.51

IVW, inverse variance weighting; WM, weighted median.

*IVW taking into account of the correlations between genetic variants was used, because the two genetic variants applied to Biobank Japan were correlated. WM was not used because of the correlation between the two SNPs.

In multivariable MR in men, in addition to the 343 genetic variants for SHBG in men, we included 125 SNPs predicting bioavailable testosterone. After excluding 3 duplicates, 8 correlated variants, and 7 with unclear correlation information, 450 genetic variants remained and were used. Genetically predicted SHBG was still associated with lower risk of IHD after controlling for bioavailable testosterone in multivariable MR (Table 3). The conditional F-statistics for SHBG and testosterone were 21.3 and 9.5 respectively. The Cochrane’s Q statistic p-value was less than 0.05, but multivariable MR Egger did not indicate pleiotropy (intercept *p* value > 0.05). The estimates were robust to using multivariable MR Egger orientated on SHBG and to using the multivariable MR median (Table 3).

**Table 3:** Associations of genetically predicted SHBG with ischemic heart disease in the UK Biobank using multivariable MR controlling for bioavailable testosterone in men.

Methods	OR	95% CI	*p*	MVMR Egger intercept *p*
MVMR IVW	0.79	0.71, 0.88	1.1 × 10^–5^	
MVMR median	0.83	0.73, 0.95	0.005	
MVMR Egger	0.83	0.72, 0.94	0.005	0.19

## Discussion

Using MR to obtain unconfounded estimates, our study suggests genetically predicted higher SHBG is associated with lower risk of IHD in men, independent of bioavailable testosterone. Our findings are not consistent with observational studies showing a positive or null association of SHBG with cardiovascular events in men^16,17^. However, SHBG increases with age, and might be affected by lifestyle and socioeconomic position, for example, SHBG is higher in smokers and those with less education^21,22^, so it is difficult to tell whether the observed positive association of SHBG with cardiovascular events is due to residual confounding in conventional observational studies. This MR study adds by providing unconfounded associations using sex-specific predictors of SHBG in a well-powered study, and also adds by showing the sex-specific role of SHBG independent of testosterone.

Our findings suggest that SHBG may play a role independent of testosterone, i.e., it might be more than a transporter of sex hormones. Given the limited mechanistic studies on the role of SHBG, the underlying pathways are unclear. SHBG suppresses inflammatory biomarkers, such as TNF-α in in vitro experiments, and the effect is not altered by co-supplementation with testosterone or estradiol^58^. However, the cardiovascular effects of TNF-α antagonism have not been confirmed in randomized controlled trials^59^. In addition, insulin resistance has recently been identified to affect IHD sex-specifically^60^. Previous MR studies suggest SHBG lowers the risk of type 2 diabetes and possibly lowers insulin resistance^26–28^, so insulin might underlie the protective effect of SHBG in IHD. Assessing these pathways and exploring other potential pathways, especially those with sex-specific responses to SHBG or with sex-specific associations with IHD, would be valuable.

Although MR studies can minimize confounding, our study has several limitations. First, MR assesses lifetime effects of an endogenous exposure rather than the short-term effects of an intervention. As such, the effect on IHD might not be comparable to the effect of interventions increasing SHBG. Second, as the genetic variants only predict a small proportion of the variance in serum SHBG, MR studies require large sample sizes^53^, which was achieved by using the UK Biobank. As such, we cannot exclude the possibility that SHBG lowers IHD in women, the null association might be because the effect size in women is smaller than what we can detect in the current study. Replication in a larger sample of women would be worthwhile. Similarly, the estimate in Japanese men had wider confidence interval than that in Europeans, possibly due to the smaller number of cases and correspondingly lower power in Japanese. Replication in a larger study of Asians is needed. Third, MR studies rely on three assumptions. Although these assumptions, especially the assumption on pleiotropy, are difficult to guarantee, we obtained consistent findings using different analytic methods with different assumptions, and using different genetic instruments, which provides confidence in the findings. Fourth, SHBG was measured at a single time point in the UK Biobank, so measurement error might exist. However, any measurement error is expected to be non-differential, thus bias is towards the null. Fifth, the genetic associations with SHBG and with IHD were both obtained from the UK Biobank, where weak instruments may bias the estimates of MR with overlapping samples^52^. However, using non-overlapping samples in the UK Biobank gave consistent estimates. Moreover, weak instrument bias is not expected given the large F-statistics (average F-statistic 176.8 in men and 120.8 in women). A recent simulation study also supports the validity of using one-sample MR in large cohort^61^, such as UK Biobank. The validity of using one-sample MR in large cohort was also supported in the recent simulation study^61^. The association was also shown for genetic variants in the *SHBG* gene, which are functionally relevant to SHBG. Sixth, the associations in Europeans may not apply to other populations. However, no reason is known for the causal effect to vary by setting, and the association with IHD was similar in Japanese men. Finally, in UK Biobank, most women are post-menopausal. Given SHBG is lower after the menopause^4^, the genetic association with SHBG in women might be underestimated, therefore the MR estimates in women might be overestimated.

From the perspective of clinical and public health practice, our findings suggest that SHBG might serve as a potential target for IHD treatment, especially in men. Dietary factors, medications, or lifestyle, such as calorie restriction^62^, that modulate SHBG might be effective for the prevention and treatment of IHD, especially in men. Identifying factors increasing SHBG and examining the pathways underlying the actions of SHBG may also provide novel insights to new drug development and drug repositioning for IHD.

## Conclusions

Genetically predicted SHBG was associated with lower risk of IHD in men, and the role of SHBG in men is independent of testosterone. Our findings suggest that SHBG might be a promising target of intervention in IHD, especially in men. Identifying factors modulating SHBG and assessing the underlying pathways could provide new insights into IHD intervention strategies, especially where the need is greatest, i.e., for men.

## Supplementary Information

Supplementary Information 1.

Supplementary Information 2.

## Data Availability

The access of data from the UK Biobank can be obtained by application to the UK Biobank (http://biobank.ctsu.ox.ac.uk/crystal/). Summary statistics for checking the association with potential confounders and pleiotropy can be downloaded from the website (http://www.nealelab.is/uk-biobank/).
