# Supplementary material for: Genetically predicted sex hormone binding globulin and ischemic heart disease in men and women: a univariable and multivariable Mendelian randomization study
Source: Sci Rep. 2021 Nov 30;11:23172. doi: 10.1038/s41598-021-02510-w (PMC8632960; doi:10.1038/s41598-021-02510-w)

Supplementary Tables

Supplementary Table S1. Data sources for summary statistics of SHBG, testosterone and ischemic heart disease used in the analysis

Supplementary Table S2. Genetic predictors for SHBG in men

Supplementary Table S3. Genetic predictors for SHBG in women

Supplementary Table S4. Associations of genetic predictors for SHBG with potential confounders in men

Supplementary Table S5. Associations of genetic predictors for SHBG with potential confounders in women

Supplementary Table S6. Sex-specific associations of genetic predictors for SHBG with LDL-cholesterol and lipoprotein (a)

Supplementary Table S7. Sex-specific associations of genetically predicted SHBG with ischemic heart disease in the UK Biobank excluding genetic variants related to BMI in univariable MR

Supplemental Table S8. Sex-specific associations of genetically predicted SHBG with ischemic heart disease in the UK Biobank using non-overlapping samples in the UK Biobank

Supplementary Figures

Supplementary Figure S1. Flow chart of the study design

Supplementary Figure S2. Scatter plot on the genetic association with sex hormone binding globulin and with ischemic heart disease in men and women in the UK Biobank

Supplementary Figure S3. Leave-one-out analysis on the sex-specific association of genetically predicted sex hormone binding globulin with ischemic heart disease in the UK Biobank

Supplementary Table 1. Data sources for summary statistics of SHBG, testosterone and ischemic heart disease used in the analysis

Phenotype	Ancestry	Sample size	Source (cohort)	PubMed ID or link
SHBG	Europeans	180,726 men and 189,473 women	UK Biobank	32042192
Testosterone	Europeans	178,782 men	UK Biobank	32042192
SHBG	Japanese	901 young men in the discovery cohort and 786 men in the validation cohort	Two Japanese cohorts	30895971
IHD	Europeans	25,409 IHD cases and 154,502 controls in white British men; 12,511 IHD cases and 199, 563 controls in white British women	UK Biobank	Accessed by application via UK Biobank
IHD	Japanese	21,611 IHD cases and 87,736 controls in men and 7,708 cases and 95,398 controls in women	Biobank Japan	http://jenger.riken.jp/en/result

Supplementary Table 2. Genetic predictors for SHBG in men

SNP	Effect Allele	Association with SHBG		Note
		Beta-coefficient	SE	
1:1510035_GGC_G	G	0.01	0.001	
1:167851935_TGTAGTGAGAGGTAGCCCAGC_T	T	0.007	0.001	
1:196914907_AGT_A	AGT	0.01	0.001	
1:23747996_GA_G	G	0.008	0.001	
1:25827633_CT_C	CT	0.011	0.001	Also associated with LDL-c, dropped in the main analysis
1:35734986_AAGTGCATCTTT_A	A	0.01	0.002	
1:93033928_CT_C	C	0.013	0.001	
11:65227616_CAAA_C	CAAA	0.007	0.001	
12:93632423_CA_C	CA	0.012	0.002	
13:115013423_TTCTC_T	TTCTC	0.009	0.001	
14:25948829_AAC_A	AAC	0.012	0.001	Also associated with BMI, and dropped in sensitivity analysis
16:1786202_TGTGTGACCATCC_T	T	0.009	0.002	
17:16062400_TA_T	T	0.006	0.001	
17:67080811_CT_C	C	0.008	0.001	
17:8567107_CA_C	CA	0.043	0.008	
19:7223973_TTTG_T	TTTG	0.011	0.001	
2:111925731_CTTATGTT_C	CTTATGTT	0.016	0.003	
2:191559843_GT_G	GT	0.01	0.001	
3:49562992_CT_C	CT	0.01	0.001	
4:128987952_ATT_A	A	0.006	0.001	
5:36206855_GAT_G	G	0.02	0.004	
6:27223966_CTCTA_C	C	0.013	0.002	
6:28419903_GGT_G	G	0.007	0.001	
7:74298343_TGAGA_T	T	0.008	0.001	
8:81430449_AT_A	A	0.022	0.002	
8:81457499_CA_C	C	0.014	0.002	
9:112204335_AT_A	AT	0.01	0.002	
9:123780856_AAAGGAAGG_A	AAAGGAAGG	0.008	0.001	
9:125949547_TA_T	TA	0.016	0.004	
9:19084633_CT_C	C	0.009	0.001	
rs10027275	G	0.013	0.001	
rs1005421	C	0.006	0.001	
rs10069690	C	0.007	0.001	
rs10083137	A	0.02	0.003	
rs10107182	T	0.012	0.001	Also associated with LDL-c, dropped in the main analysis
rs10116426	C	0.009	0.001	
rs10153800	A	0.007	0.001	
rs1037169	T	0.012	0.001	

rs10411958	C	0.007	0.001
rs10421262	T	0.011	0.001
rs1058319	T	0.009	0.002
rs10649697	T	0.009	0.001
rs10657979	C	0.016	0.001
rs10822153	A	0.064	0.001
rs10864086	C	0.015	0.001
rs10868080	T	0.021	0.001
rs10871794	A	0.006	0.001
rs10895277	A	0.01	0.001
rs11111274	G	0.007	0.001
rs111643014	A	0.024	0.004
rs111767734	T	0.032	0.006
rs111917063	C	0.019	0.004
rs111981233	G	0.026	0.002
rs112352679	T	0.01	0.002
rs112965849	A	0.009	0.002
rs11398692	C	0.006	0.001
rs114165349	G	0.087	0.004
rs11419346	C	0.006	0.002
rs114949263	C	0.019	0.002
rs11539938	C	0.01	0.001
rs11601507	A	0.016	0.002
rs11621792	C	0.013	0.001
rs116338429	T	0.01	0.002
rs11641834	C	0.012	0.001
rs116483731	A	0.05	0.007
rs11655704	C	0.031	0.001
rs11666245	G	0.016	0.003
rs11732763	A	0.012	0.002
rs11734408	G	0.008	0.001
rs11739158	T	0.008	0.001
rs11743810	T	0.009	0.001
rs11751920	C	0.023	0.004
rs11856926	G	0.012	0.001
rs11867902	G	0.021	0.004
rs11994858	G	0.011	0.001
rs12059956	G	0.008	0.001
rs1229492	T	0.011	0.001
rs12364060	T	0.008	0.001
rs12454712	C	0.006	0.001
rs12536766	T	0.005	0.001
rs12543287	C	0.011	0.001

rs1260326	C	0.038	0.001	Also associated with alcohol drinking and LDL-c, dropped in the main analysis
rs12690320	G	0.005	0.001	
rs12694450	T	0.007	0.001	
rs12696304	G	0.008	0.001	
rs12705095	G	0.017	0.002	
rs12797706	A	0.012	0.001	
rs12818938	T	0.009	0.002	
rs12928099	A	0.009	0.001	
rs12950562	T	0.011	0.001	
rs13042148	C	0.012	0.002	
rs13108218	A	0.023	0.001	
rs13280055	G	0.008	0.002	
rs13315174	G	0.007	0.001	
rs13379043	C	0.008	0.001	
rs13389219	T	0.011	0.001	
rs1349852	C	0.02	0.001	
rs1351394	T	0.007	0.001	
rs138529890	A	0.025	0.003	
rs139974673	T	0.074	0.004	
rs140386498	T	0.032	0.005	
rs140584594	A	0.012	0.001	
rs141811210	GTAA	0.007	0.001	
rs1418334	G	0.04	0.001	Also associated with LDL-c, dropped in the main analysis
rs142627977	C	0.012	0.001	
rs142740991	C	0.008	0.001	
rs143709973	A	0.014	0.003	
rs144647926	A	0.012	0.002	
rs144794875	G	0.01	0.002	
rs148082013	G	0.054	0.01	
rs148427769	G	0.014	0.003	
rs148911629	G	0.044	0.006	
rs149624078	C	0.098	0.005	
rs149663666	T	0.047	0.007	
rs149675684	G	0.079	0.013	
rs1567353	C	0.006	0.001	
rs1570516	T	0.006	0.001	
rs157935	G	0.012	0.001	
rs1640267	C	0.017	0.001	
rs17036326	G	0.017	0.002	
rs17050272	A	0.006	0.001	Also associated with LDL-c, dropped in the main analysis
rs17145750	T	0.011	0.002	
rs17185536	T	0.005	0.001	

rs1730865	G	0.026	0.001	
rs174528	T	0.008	0.001	Also associated with LDL-c, dropped in the main analysis
rs17583875	A	0.024	0.004	
rs17669311	G	0.01	0.001	
rs17747324	T	0.006	0.001	
rs1782652	T	0.013	0.001	
rs1788641	A	0.007	0.001	
rs1799831	C	0.009	0.002	
rs1799941	A	0.121	0.001	
rs1801689	A	0.033	0.004	Also associated with LDL-c, dropped in the main analysis
rs180956060	G	0.013	0.002	
rs184640919	G	0.096	0.019	
rs185073832	C	0.106	0.017	
rs1859690	G	0.015	0.002	
rs186915841	C	0.094	0.015	
rs1870927	A	0.006	0.001	
rs1871395	A	0.033	0.002	
rs187325356	A	0.065	0.007	
rs187437	G	0.008	0.001	
rs1883783	T	0.006	0.001	
rs1890426	C	0.007	0.001	
rs190712692	A	0.014	0.003	Also associated with LDL-c, dropped in the main analysis
rs191591035	G	0.102	0.014	
rs191884522	C	0.13	0.015	
rs198384	A	0.007	0.001	
rs1991401	G	0.007	0.001	
rs199910997	A	0.01	0.001	
rs200138078	C	0.007	0.001	Also associated with BMI, and dropped in sensitivity analysis
rs2002094	C	0.012	0.002	
rs200883214	G	0.006	0.001	
rs201764793	TTTC	0.009	0.001	
rs202128511	T	0.013	0.001	
rs202200760	C	0.071	0.003	
rs2106727	G	0.005	0.001	
rs2106854	T	0.008	0.002	
rs211644	C	0.006	0.001	
rs2156805	G	0.005	0.001	
rs2197771	G	0.006	0.001	
rs2216707	C	0.008	0.002	
rs2222018	C	0.013	0.001	
rs2234694	A	0.013	0.003	
rs2238799	G	0.007	0.001	

rs2239222	G	0.011	0.001	
rs2247213	G	0.014	0.001	
rs2259305	G	0.012	0.001	
rs2275560	G	0.007	0.002	
rs2277283	C	0.008	0.001	
rs2283760	C	0.005	0.001	
rs2287322	G	0.019	0.001	
rs2288004	G	0.007	0.001	
rs234051	A	0.008	0.001	
rs2431752	A	0.01	0.002	
rs246192	G	0.009	0.001	
rs2564923	A	0.008	0.001	
rs2721195	T	0.011	0.001	
rs2820441	C	0.007	0.001	
rs28507491	A	0.014	0.001	
rs28562483	G	0.011	0.002	
rs28650012	G	0.007	0.001	
rs28929474	T	0.138	0.004	
rs2905801	T	0.014	0.001	
rs29681	T	0.009	0.002	
rs3006593	C	0.008	0.001	
rs329122	G	0.006	0.001	
rs34006916	G	0.017	0.003	
rs34255979	T	0.027	0.002	Also associated with LDL-c, dropped in the main analysis
rs34372369	A	0.022	0.003	
rs34390319	C	0.015	0.002	
rs34651	T	0.01	0.002	
rs34771269	CT	0.012	0.001	
rs35067979	C	0.017	0.002	
rs35225944	T	0.01	0.001	
rs35234337	C	0.008	0.001	
rs35526088	T	0.007	0.001	
rs35654957	T	0.008	0.001	
rs35824797	C	0.016	0.002	
rs36013981	A	0.007	0.001	
rs36086195	T	0.017	0.001	
rs36103835	T	0.008	0.001	
rs36108764	G	0.01	0.002	
rs370654105	C	0.007	0.001	
rs3742366	C	0.009	0.001	
rs3746575	G	0.017	0.001	
rs3747647	C	0.008	0.002	

rs3780190	G	0.013	0.001	
rs3781085	T	0.006	0.001	
rs3782735	A	0.007	0.001	
rs3795128	C	0.01	0.001	
rs3812275	C	0.007	0.001	
rs3927496	A	0.01	0.001	
rs40270	A	0.014	0.001	
rs4077285	G	0.012	0.002	
rs41284816	T	0.02	0.005	
rs41412647	C	0.01	0.001	
rs4333851	A	0.008	0.002	
rs445	C	0.014	0.002	
rs45490496	A	0.009	0.001	
rs45512696	T	0.021	0.002	
rs4645936	G	0.015	0.003	
rs4673528	C	0.006	0.001	
rs4675682	T	0.009	0.001	
rs4714001	G	0.005	0.001	
rs4715316	T	0.009	0.001	
rs4725944	C	0.009	0.001	
rs4762755	T	0.007	0.001	
rs4782568	G	0.015	0.001	
rs4809604	T	0.006	0.001	
rs4812336	A	0.008	0.001	
rs4841133	G	0.022	0.002	Also associated with LDL-c, dropped in the main analysis
rs4860987	T	0.03	0.001	Also associated with LDL-c, dropped in the main analysis
rs4979372	C	0.013	0.001	
rs4988308	G	0.005	0.001	
rs501470	G	0.015	0.001	Also associated with lipoprotein (a), dropped in the main analysis
rs528350911	C	0.042	0.008	
rs540730	T	0.018	0.001	
rs549677458	A	0.1	0.018	
rs55646464	G	0.007	0.001	
rs55855238	C	0.009	0.001	
rs55962409	C	0.008	0.001	
rs56187480	G	0.013	0.001	
rs56196860	A	0.022	0.004	
rs56237852	C	0.007	0.002	
rs562609617	GT	0.011	0.001	
rs56332871	A	0.03	0.001	
rs565471584	T	0.078	0.012	
rs565728741	C	0.012	0.002	

rs5749082	T	0.009	0.001	
rs58321169	C	0.008	0.001	
rs591939	A	0.009	0.001	
rs59708846	A	0.019	0.002	
rs60005573	GGATTAT	0.008	0.002	
rs60018147	G	0.013	0.002	
rs6005840	A	0.017	0.001	
rs61755050	T	0.129	0.008	
rs61929307	G	0.007	0.001	
rs62012358	G	0.007	0.001	
rs62134282	T	0.007	0.001	
rs62182125	G	0.006	0.001	
rs62195072	C	0.008	0.001	
rs62394490	T	0.011	0.001	
rs62442919	A	0.007	0.001	
rs6258	C	0.61	0.007	
rs62580766	T	0.01	0.002	
rs62618693	T	0.019	0.003	
rs631695	T	0.017	0.001	
rs6480299	C	0.011	0.001	
rs6595447	T	0.008	0.002	
rs6632893	A	0.006	0.001	
rs665731	T	0.007	0.002	
rs6736913	A	0.033	0.004	
rs6750410	A	0.018	0.002	
rs67527887	T	0.007	0.001	
rs6792725	G	0.012	0.001	
rs6831352	T	0.02	0.001	
rs687339	C	0.028	0.001	Also associated with BMI, and dropped in sensitivity analysis
rs6900473	A	0.009	0.001	
rs6939861	G	0.011	0.001	
rs6950023	G	0.032	0.002	
rs6965401	G	0.014	0.003	
rs7096937	T	0.009	0.001	
rs71115649	A	0.008	0.001	
rs71584764	TA	0.01	0.002	
rs71586027	TATCTC	0.01	0.001	
rs7175361	A	0.009	0.002	
rs7210574	C	0.01	0.001	
rs7254776	C	0.005	0.001	Also associated with LDL-c, dropped in the main analysis
rs72663907	T	0.012	0.002	
rs72683923	C	0.034	0.004	

rs72708162	T	0.016	0.002	
rs72729610	A	0.01	0.002	
rs72753908	C	0.011	0.002	
rs7286550	G	0.008	0.001	
rs72948115	C	0.012	0.002	
rs73109480	T	0.014	0.002	
rs7314285	G	0.03	0.002	
rs73266316	T	0.013	0.002	
rs73547906	C	0.006	0.001	
rs73631501	T	0.011	0.001	
rs738409	G	0.031	0.001	
rs73909848	G	0.014	0.002	
rs7438888	C	0.071	0.009	
rs749761903	A	0.008	0.001	
rs75130744	G	0.03	0.002	
rs75170914	T	0.012	0.001	
rs75349541	C	0.009	0.002	
rs753645751	G	0.009	0.002	
rs7540115	C	0.009	0.002	
rs754959778	CGT	0.005	0.001	
rs757253787	T	0.008	0.001	
rs7623513	C	0.009	0.002	
rs762450647	CT	0.007	0.001	
rs7631981	G	0.007	0.001	
rs7655064	T	0.008	0.002	
rs768159759	A	0.013	0.002	
rs768651403	GT	0.008	0.001	
rs76895963	G	0.055	0.005	Also associated with BMI, and dropped in sensitivity analysis
rs7694379	G	0.021	0.001	
rs769447487	T	0.011	0.001	
rs771193934	AGGCATGCTGCCAAGAAT	0.006	0.001	Also associated with LDL-c, dropped in the main analysis
rs771435780	G	0.012	0.001	
rs7735249	C	0.018	0.002	
rs773816354	TAACAGGAGTTAAAAGTGTCG	0.012	0.001	
rs775181992	A	0.01	0.001	
rs7780562	C	0.008	0.002	
rs778239811	T	0.006	0.001	
rs778571122	GTGTTTTTTTTGTTTTTGTTTT	0.01	0.001	
rs780309846	AAAAAAC	0.005	0.001	
rs7808613	C	0.008	0.001	

rs78319058	T	0.025	0.004
rs78444298	G	0.026	0.004
rs78890745	A	0.014	0.002
rs7892835	G	0.008	0.001
rs79287178	G	0.04	0.004
rs79354983	A	0.016	0.002
rs79717793	G	0.025	0.002
rs8030357	G	0.01	0.001
rs8038032	G	0.011	0.001
rs8038465	T	0.006	0.001
rs8066941	T	0.015	0.001
rs8176526	C	0.006	0.001
rs820503	C	0.011	0.002
rs856534	A	0.01	0.001
rs860262	A	0.01	0.001
rs864899	A	0.009	0.001
rs876435	G	0.008	0.001
rs904801	A	0.01	0.001
rs907866	G	0.008	0.001
rs9266184	T	0.01	0.001
rs9379084	G	0.015	0.002
rs9697210	G	0.019	0.002
rs9738226	G	0.019	0.001
rs9914426	G	0.008	0.001
rs997907	C	0.008	0.001
X:133611871_AT_A	A	0.009	0.001

Genetic correlation between SHBG and bioavailable testosterone in men was -0.05 [1]

Supplementary Table 3. Genetic predictors for SHBG in women

SNP	Effect Allele	Association with SHBG		Note
		Beta-coefficient	SE	
1:155867257_CAA_C	C	0.009	0.002	
1:221048577_TGA_T	T	0.009	0.002	
1:23747996_GA_G	G	0.011	0.002	
1:57170378_AATC_A	A	0.011	0.002	
1:93787087_TA_T	T	0.009	0.002	
10:65158772_AAAG_A	A	0.047	0.001	
12:103483327_AT_A	AT	0.026	0.001	
12:12562340_GT_G	G	0.01	0.002	
12:26614614_CAT_C	C	0.01	0.002	
13:50565104_ACT_A	A	0.038	0.005	
15:43025006_CT_C	CT	0.117	0.018	
15:51052455_CT_C	C	0.008	0.002	
15:53094375_TTTTG_T	TTTTG	0.028	0.002	
17:48627860_CCT_C	C	0.009	0.002	
19:7223973_TTTG_T	TTTG	0.018	0.001	
2:191559843_GT_G	GT	0.011	0.002	
2:208469213_ATCTT_A	ATCTT	0.012	0.002	
3:122361173_TTTTTC_T	TTTTTC	0.01	0.002	
4:103877471_TA_T	T	0.009	0.001	
6:130386212_GGAGA_G	GGAGA	0.009	0.002	
7:130438531_CTTTTTT_C	C	0.01	0.001	
8:59415339_TTG_T	T	0.011	0.001	Also associated with LDL-c, dropped in the main analysis
8:81457499_CA_C	C	0.026	0.002	
9:127474886_CA_C	CA	0.007	0.001	
rs1005421	C	0.009	0.001	
rs10095380	G	0.01	0.002	
rs10095930	C	0.011	0.001	
rs10108150	A	0.008	0.001	
rs10153315	T	0.009	0.001	
rs10189479	A	0.007	0.001	
rs10238028	G	0.019	0.003	
rs1033667	T	0.011	0.002	
rs1037169	T	0.015	0.002	
rs10461018	T	0.01	0.001	
rs1047891	A	0.014	0.002	
rs10486782	A	0.007	0.002	
rs10489206	C	0.009	0.002	
rs10511002	A	0.008	0.002	
rs10622246	ATTTT	0.01	0.001	

rs10747689	T	0.006	0.001	
rs10774095	A	0.007	0.002	
rs10815276	G	0.009	0.001	
rs10857228	T	0.009	0.002	
rs10880868	C	0.008	0.002	
rs10883451	C	0.009	0.001	
rs10893876	C	0.009	0.002	
rs10939934	A	0.007	0.001	
rs10961205	A	0.007	0.001	
rs11021232	T	0.013	0.002	
rs11047237	A	0.03	0.004	
rs11054861	A	0.022	0.004	
rs11078597	C	0.017	0.002	
rs11079685	A	0.008	0.001	
rs11103377	G	0.008	0.001	
rs111270317	CGTGT	0.009	0.002	
rs111288118	C	0.013	0.002	
rs11130982	T	0.007	0.002	
rs11186719	A	0.012	0.001	
rs11188601	C	0.01	0.001	
rs112332688	A	0.008	0.002	
rs1126161	G	0.007	0.002	
rs1128249	T	0.017	0.001	Also associated with LDL-c, dropped in the main analysis
rs112966033	TACACAC	0.016	0.001	
rs113408476	A	0.012	0.002	
rs11376788	AC	0.008	0.002	
rs114165349	G	0.07	0.005	Also associated with LDL-c, dropped in the main analysis
rs114816312	T	0.082	0.008	
rs114949263	C	0.015	0.002	
rs115209326	T	0.076	0.012	
rs11539938	C	0.009	0.001	
rs11556924	T	0.012	0.001	
rs11601507	A	0.016	0.003	Also associated with LDL-c, dropped in the main analysis
rs11621792	C	0.025	0.001	Also associated with LDL-c, dropped in the main analysis
rs116279971	C	0.041	0.007	
rs11637595	C	0.009	0.002	
rs11641834	C	0.011	0.001	
rs11664106	T	0.006	0.002	
rs11666245	G	0.017	0.003	
rs11668201	T	0.009	0.002	
rs11688682	C	0.009	0.002	
rs11690748	C	0.007	0.001	

rs11720108	T	0.007	0.002	
rs11738093	A	0.012	0.002	
rs117522510	A	0.05	0.01	
rs11774700	C	0.006	0.002	
rs11780978	A	0.008	0.001	
rs11791747	G	0.011	0.002	
rs11830764	C	0.04	0.003	
rs11967262	C	0.009	0.001	
rs12012896	A	0.025	0.001	
rs12138803	C	0.008	0.002	
rs1223796	G	0.013	0.002	
rs12280075	G	0.008	0.002	
rs1229498	T	0.011	0.002	
rs12311848	G	0.011	0.002	Also associated with LDL-c, dropped in the main analysis
rs12438742	G	0.008	0.001	
rs12454712	C	0.008	0.001	
rs12543287	C	0.011	0.001	
rs12593818	T	0.01	0.002	
rs1260326	C	0.034	0.001	Also associated with alcohol drinking and LDL-c, dropped in the main analysis
rs12613243	T	0.015	0.003	
rs12624244	A	0.021	0.003	
rs12787996	C	0.007	0.002	
rs12797706	A	0.014	0.002	
rs12804411	T	0.015	0.002	
rs12864658	T	0.015	0.003	
rs12906447	C	0.009	0.001	
rs13018007	G	0.012	0.003	
rs13042148	C	0.014	0.002	
rs13108218	A	0.022	0.001	Also associated with LDL-c, dropped in the main analysis
rs13150068	A	0.017	0.001	
rs13200245	A	0.008	0.002	
rs13237750	C	0.017	0.003	
rs1330307	A	0.009	0.001	
rs13303359	C	0.007	0.001	
rs13379043	C	0.01	0.002	
rs13394092	C	0.008	0.002	
rs138204164	C	0.011	0.002	
rs139974673	T	0.048	0.004	
rs140302625	T	0.067	0.002	
rs140312320	G	0.016	0.003	
rs140584594	A	0.015	0.002	
rs1418652	C	0.007	0.001	

rs1433210	C	0.008	0.002	
rs143378550	A	0.02	0.005	
rs144293483	AC	0.014	0.003	
rs146104952	CCAAAAAATA	0.011	0.002	
rs147153202	G	0.013	0.002	
rs148911629	G	0.041	0.007	
rs150115323	G	0.005	0.001	
rs150584036	TAC	0.009	0.002	
rs1530439	T	0.012	0.002	
rs1634791	A	0.01	0.001	Also associated with LDL-c, dropped in the main analysis
rs1650527	C	0.011	0.002	
rs1684608	C	0.009	0.002	
rs16995626	C	0.017	0.003	
rs17128091	C	0.012	0.002	
rs17202341	G	0.008	0.001	
rs1730862	G	0.021	0.001	
rs1738386	C	0.009	0.001	
rs1741344	T	0.007	0.001	
rs174537	G	0.014	0.001	Also associated with LDL-c, dropped in the main analysis
rs17492269	G	0.009	0.002	
rs17580	A	0.023	0.003	
rs17583875	A	0.027	0.005	
rs17616365	G	0.026	0.004	
rs17669311	G	0.009	0.001	
rs1782652	T	0.012	0.001	
rs17887160	C	0.009	0.002	
rs1801282	G	0.02	0.002	
rs183015141	G	0.029	0.005	
rs185044544	T	0.021	0.005	
rs1870927	A	0.008	0.001	
rs1872992	A	0.006	0.002	
rs191591035	G	0.113	0.017	
rs1962883	C	0.006	0.001	
rs198358	C	0.006	0.002	
rs199607859	T	0.01	0.001	Also associated with LDL-c, dropped in the main analysis
rs2009310	T	0.006	0.001	
rs201468966	C	0.016	0.002	
rs2018519	C	0.019	0.002	
rs201874554	A	0.015	0.003	
rs202021413	TA	0.01	0.001	
rs202200760	C	0.079	0.004	
rs2057655	A	0.009	0.002	Also associated with LDL-c, dropped in the main analysis

rs2064074	A	0.008	0.001	
rs2068888	A	0.011	0.001	Also associated with LDL-c, dropped in the main analysis
rs2176040	A	0.015	0.001	
rs2207132	G	0.023	0.004	Also associated with LDL-c, dropped in the main analysis
rs2239222	G	0.009	0.002	
rs2246223	T	0.007	0.001	
rs2288926	A	0.005	0.002	
rs2299055	A	0.009	0.002	
rs2364717	T	0.007	0.001	
rs2438109	C	0.008	0.002	
rs2498786	C	0.013	0.001	
rs2525570	G	0.009	0.001	
rs267733	A	0.014	0.002	
rs2723555	T	0.006	0.001	
rs275177	C	0.009	0.002	
rs28360642	A	0.02	0.002	
rs28459049	C	0.007	0.002	Also associated with LDL-c, dropped in the main analysis
rs28636815	G	0.011	0.001	
rs28712547	G	0.01	0.002	
rs28925904	C	0.028	0.005	
rs28929470	A	0.054	0.011	
rs28929474	T	0.063	0.005	
rs2915023	G	0.01	0.002	
rs2924808	C	0.007	0.001	
rs2925979	C	0.013	0.002	
rs2970871	T	0.008	0.001	
rs2980858	C	0.008	0.002	Also associated with LDL-c, dropped in the main analysis
rs2986669	A	0.009	0.002	
rs3001032	C	0.012	0.002	
rs3018695	A	0.008	0.001	
rs34154818	A	0.009	0.001	
rs34184867	C	0.008	0.001	
rs34255979	T	0.027	0.002	Also associated with LDL-c, dropped in the main analysis
rs34311866	T	0.01	0.002	
rs34331968	T	0.012	0.001	
rs34385891	AT	0.008	0.001	
rs34499031	T	0.009	0.002	
rs34651	T	0.009	0.003	
rs35143646	T	0.008	0.002	
rs35198068	T	0.008	0.002	Also associated with BMI, dropped in the sensitivity analysis
rs35233014	C	0.014	0.002	

rs35475471	CAT	0.008	0.002	
rs35568851	CT	0.008	0.001	Also associated with LDL-c, dropped in the main analysis
rs35696875	TCA	0.011	0.002	
rs3733321	T	0.007	0.001	
rs3747207	A	0.019	0.002	
rs3747367	A	0.007	0.002	
rs3749228	C	0.013	0.003	
rs3751129	A	0.01	0.002	
rs3768420	C	0.008	0.002	
rs3782735	A	0.011	0.001	
rs390408	A	0.015	0.002	
rs40270	A	0.019	0.002	
rs4077285	G	0.014	0.002	
rs4092465	G	0.011	0.001	
rs4122352	A	0.011	0.002	
rs41280463	G	0.01	0.002	
rs4149056	T	0.027	0.002	
rs4264433	T	0.019	0.001	Also associated with LDL-c, dropped in the main analysis
rs4307773	T	0.013	0.001	
rs4327143	A	0.008	0.002	
rs4450871	G	0.008	0.001	
rs4530527	C	0.008	0.001	
rs4563785	G	0.014	0.003	
rs469721	C	0.013	0.002	
rs4709746	T	0.009	0.002	
rs4804669	A	0.009	0.002	
rs4810580	T	0.01	0.002	
rs4830411	G	0.008	0.001	
rs4837794	T	0.012	0.002	
rs4871015	A	0.007	0.001	
rs4876993	T	0.008	0.001	
rs4976033	A	0.009	0.001	
rs5112	G	0.009	0.002	Also associated with LDL-c, dropped in the main analysis
rs5117	C	0.012	0.002	Also associated with LDL-c, dropped in the main analysis
rs528350911	C	0.058	0.01	
rs528806375	A	0.009	0.001	
rs545206972	C	0.69	0.009	
rs548235873	ATTT	0.007	0.002	
rs549664712	C	0.013	0.002	
rs550767741	ATTTTTTTTTTTTTTTTTTTTTTTTTTTTTTTT	0.022	0.002	
rs555234816	T	0.017	0.003	

rs555754	A	0.016	0.001	Also associated with LDL-c, dropped in the main analysis
rs560436873	A	0.119	0.018	
rs56332871	A	0.038	0.002	
rs568656	C	0.011	0.002	
rs57158761	A	0.007	0.001	
rs5750131	G	0.006	0.001	
rs5753111	T	0.014	0.002	
rs5791099	TA	0.006	0.002	
rs5813220	G	0.013	0.002	
rs5820605	CT	0.007	0.001	
rs58321169	C	0.008	0.002	
rs58489806	C	0.011	0.003	Also associated with LDL-c, dropped in the main analysis
rs59774409	T	0.016	0.003	
rs59837038	T	0.008	0.002	
rs60018147	G	0.014	0.002	
rs6058067	G	0.009	0.002	
rs6073431	T	0.017	0.001	
rs6088776	T	0.012	0.002	
rs6129778	A	0.013	0.002	Also associated with LDL-c, dropped in the main analysis
rs61755050	T	0.13	0.009	
rs61830291	C	0.014	0.002	
rs62128735	A	0.009	0.002	
rs62186584	C	0.007	0.002	
rs62271373	T	0.022	0.003	
rs62303689	C	0.009	0.002	
rs62486442	G	0.007	0.002	
rs62515079	G	0.031	0.005	
rs62580766	T	0.011	0.002	
rs6531735	G	0.006	0.001	
rs6546096	A	0.028	0.002	
rs6706	T	0.018	0.002	
rs6736913	A	0.034	0.005	
rs67651018	A	0.008	0.002	
rs6772177	C	0.012	0.002	
rs67890964	C	0.009	0.001	
rs6792725	G	0.017	0.002	
rs68002561	G	0.013	0.002	
rs6803518	T	0.007	0.002	
rs6831257	G	0.008	0.001	
rs6860245	C	0.011	0.002	
rs687339	C	0.036	0.002	Also associated with BMI, dropped in the sensitivity analysis
rs6879874	T	0.008	0.002	

rs696825	T	0.025	0.002	
rs7139079	A	0.013	0.001	Also associated with LDL-c, dropped in the main analysis
rs71468663	A	0.017	0.003	
rs71531849	CT	0.011	0.002	
rs7250869	C	0.01	0.002	
rs7252372	G	0.007	0.001	
rs72648854	C	0.018	0.004	
rs72681869	C	0.059	0.007	
rs72766607	T	0.027	0.005	
rs72782727	G	0.009	0.002	
rs72836346	C	0.013	0.003	
rs72844546	C	0.01	0.001	
rs7298820	T	0.014	0.002	
rs73036519	G	0.012	0.002	
rs7321688	C	0.007	0.002	
rs73519353	A	0.075	0.013	
rs73670309	C	0.011	0.002	
rs740516	C	0.009	0.002	Also associated with LDL-c, dropped in the main analysis
rs740893	G	0.009	0.002	
rs74090351	A	0.016	0.003	
rs7475279	A	0.021	0.002	
rs7481219	A	0.009	0.002	
rs7484541	T	0.014	0.002	
rs75077113	C	0.01	0.002	
rs75130744	G	0.026	0.003	
rs7567544	C	0.008	0.001	
rs764029425	TG	0.038	0.002	
rs76491020	C	0.011	0.003	
rs76767219	A	0.047	0.004	
rs768159759	A	0.014	0.003	
rs76895963	G	0.073	0.005	
rs7696472	A	0.006	0.001	
rs770971500	C	0.01	0.002	
rs771193934	AGGCATGCTG CCAAGAAT	0.01	0.002	Also associated with LDL-c, dropped in the main analysis
rs775181992	A	0.014	0.002	
rs778571122	GTGTTTTTTTT GTTTTTGTTTT	0.011	0.001	
rs78057960	T	0.009	0.002	
rs78058190	G	0.023	0.004	
rs781996653	TTATTTTATTG	0.009	0.001	
rs784504	C	0.01	0.002	
rs78890745	A	0.022	0.002	
rs79237700	T	0.019	0.004	

rs79287178	G	0.028	0.004	
rs79391862	A	0.074	0.006	
rs799157	T	0.021	0.003	
rs80126506	A	0.008	0.001	
rs80235628	G	0.021	0.003	
rs8027064	A	0.026	0.004	
rs8107967	G	0.01	0.001	
rs8176741	G	0.016	0.003	
rs8178824	C	0.042	0.004	Also associated with LDL-c, dropped in the main analysis
rs820504	G	0.013	0.002	
rs848476	G	0.007	0.002	
rs858519	C	0.1	0.001	
rs868655	C	0.008	0.002	
rs8756	C	0.009	0.001	
rs892225	G	0.007	0.001	
rs899865	T	0.006	0.002	
rs921153	A	0.009	0.002	
rs925098	G	0.01	0.002	
rs9366291	C	0.008	0.001	
rs9379084	G	0.015	0.002	
rs9426829	C	0.014	0.001	
rs9461793	A	0.009	0.002	
rs9556403	G	0.007	0.001	
rs9644032	T	0.006	0.001	
rs9697210	G	0.016	0.002	
rs9834503	A	0.008	0.001	
rs9872754	C	0.009	0.002	
rs9987289	G	0.022	0.002	Also associated with LDL-c, dropped in the main analysis

Genetic correlation between SHBG and bioavailable testosterone in women was -0.74 [1]

Supplementary Table 4. Associations of genetic predictors for SHBG with potential confounders in men

SNP	P values with potential confounders				
	Moderate physical activity	Vigorous physical activity	Townsend index	smoking	Alcohol drinking
rs3742366	0.865	0.853	0.034	0.00002	0.041
rs1418334	0.036	0.019	0.077	0.000	0.358
rs6831352	0.861	0.605	0.190	0.001	0.043
rs329122	0.114	0.624	0.033	0.001	0.683
rs200138078	0.168	0.968	0.010	0.001	0.446
rs11419346	0.700	0.630	0.882	0.002	0.170
rs28507491	0.020	0.077	0.037	0.002	0.799
rs200883214	0.845	0.855	0.117	0.003	0.275
rs12364060	0.367	0.719	0.470	0.004	0.483
rs4675682	0.858	0.159	0.085	0.005	0.425
rs34255979	0.141	0.016	0.115	0.007	0.807
rs28929474	0.189	0.554	0.021	0.008	0.000
19:7223973_TTTG_T	0.790	0.655	0.196	0.008	0.332
rs11743810	0.510	0.178	0.207	0.008	0.889
rs4714001	0.555	0.729	0.413	0.009	0.752
rs62618693	0.299	0.417	0.296	0.009	0.920
rs72753908	0.606	0.430	0.955	0.013	0.849
rs11734408	0.905	0.112	0.772	0.013	0.074
rs59708846	0.330	0.178	0.571	0.014	0.551
rs3795128	0.110	0.483	0.059	0.015	0.006
rs11751920	0.289	0.820	0.261	0.021	0.026
rs6750410	0.211	0.566	0.193	0.023	0.073
rs34651	0.171	0.285	0.030	0.023	0.449
rs62012358	0.367	0.121	0.913	0.023	0.000
rs10868080	0.640	0.905	0.072	0.028	0.100
rs4988308	0.783	0.667	0.192	0.028	0.289
rs62580766	0.029	0.398	0.248	0.029	0.292
rs7286550	0.124	0.029	0.765	0.031	0.098
rs445	0.179	0.892	0.483	0.032	0.055
rs55646464	0.285	0.686	0.374	0.034	0.640
rs4841133	0.323	0.562	0.818	0.039	0.725
rs7540115	0.097	0.251	0.408	0.045	0.642
rs112965849	0.955	0.655	0.737	0.045	0.908
rs190712692	0.879	0.293	0.914	0.046	0.004
rs6005840	0.417	0.201	0.259	0.051	0.330
rs149624078	0.946	0.653	0.459	0.054	0.026
rs34006916	0.834	0.572	0.743	0.054	0.288
rs749761903	0.482	0.442	0.067	0.056	0.055
rs45512696	0.847	0.350	0.867	0.058	0.900
rs12950562	0.774	0.279	0.057	0.060	0.072

rs7631981	0.109	0.817	0.207	0.061	0.694
8:81430449_AT_A	0.096	0.240	0.150	0.061	0.177
17:16062400_TA_T	0.673	0.702	0.028	0.062	0.053
14:25948829_AAC_A	0.741	0.624	0.658	0.064	0.688
rs234051	0.578	0.929	0.511	0.066	0.431
rs10822153	0.379	0.877	0.145	0.069	0.288
6:28419903_GGT_G	0.604	0.019	0.150	0.070	0.335
rs1801689	0.561	0.384	0.858	0.070	0.333
rs4762755	0.228	0.168	0.566	0.070	0.324
rs78890745	0.859	0.438	0.176	0.071	0.423
rs140386498	0.476	0.591	0.292	0.073	0.652
rs36013981	0.794	0.814	0.471	0.078	0.020
rs2283760	0.962	0.285	0.023	0.079	0.169
rs5749082	0.456	0.631	0.987	0.080	0.914
1:167851935_TGTAGTGAGAGGTAGCCCAGC_T	0.912	0.008	0.700	0.082	0.957
rs9914426	0.029	0.618	0.642	0.096	0.523
rs10864086	0.327	0.133	0.585	0.104	0.186
rs4809604	0.085	0.295	0.398	0.106	0.783
rs738409	0.140	0.256	0.039	0.109	0.352
rs1058319	0.558	0.215	0.773	0.110	0.101
rs73109480	0.279	0.894	0.170	0.118	0.317
rs2288004	0.744	0.250	0.004	0.118	0.000
rs778571122	0.656	0.953	0.529	0.118	0.339
rs141811210	0.284	0.352	0.364	0.121	0.937
rs13280055	0.023	0.243	0.355	0.122	0.079
rs34372369	0.479	0.039	0.170	0.124	0.607
17:8567107_CA_C	0.557	0.833	0.720	0.126	0.916
rs8038032	0.579	0.389	0.750	0.128	0.061
rs10069690	0.212	0.561	0.344	0.130	0.810
rs2277283	0.061	0.730	0.414	0.139	0.584
rs4673528	0.049	0.906	0.803	0.140	0.268
rs2287322	0.286	0.897	0.754	0.142	0.183
rs17036326	0.575	0.037	0.619	0.143	0.952
rs58321169	0.646	0.611	0.865	0.146	0.833
rs149675684	0.732	0.380	0.976	0.149	0.010
rs35526088	0.638	0.823	0.585	0.150	0.954
rs7254776	0.552	0.893	0.504	0.153	0.014
rs1859690	0.549	0.106	0.376	0.155	0.557
rs1883783	0.179	0.363	0.341	0.155	0.608
rs13315174	0.341	0.272	0.950	0.160	0.282
rs4725944	0.485	0.886	0.388	0.167	0.663
rs10421262	0.319	0.724	0.779	0.168	0.534
rs62182125	0.000001	0.001	0.039	0.173	0.490
rs140584594	0.952	0.658	0.836	0.179	0.004

rs907866	0.200	0.784	0.301	0.179	0.501
7:74298343_TGAGA_T	0.581	0.265	0.351	0.179	0.001
rs55962409	0.066	0.065	0.044	0.180	0.379
rs111981233	0.635	0.721	0.292	0.181	0.144
rs36108764	0.583	0.810	0.754	0.182	0.627
rs3781085	0.573	0.558	0.076	0.183	0.275
rs860262	0.594	0.369	0.990	0.184	0.455
rs6950023	0.895	0.358	0.392	0.184	0.630
rs2002094	0.586	0.080	0.765	0.186	0.018
rs6632893	0.989	0.018	0.104	0.188	0.214
rs562609617	0.977	0.005	0.775	0.189	0.788
rs111917063	0.240	0.675	0.455	0.191	0.012
rs3746575	0.213	0.964	0.095	0.202	0.252
rs864899	0.062	0.981	0.212	0.207	0.990
rs10027275	0.009	0.394	0.633	0.210	0.054
rs34390319	0.445	0.690	0.085	0.211	0.517
rs12536766	0.256	0.617	0.920	0.213	0.730
rs142740991	0.314	0.051	0.259	0.213	0.426
rs17145750	0.564	0.516	0.812	0.221	0.018
rs631695	0.535	0.995	0.579	0.225	0.754
rs12694450	0.152	0.566	0.047	0.226	0.398
rs187437	0.366	0.262	0.624	0.229	0.009
rs17050272	0.903	0.936	0.963	0.229	0.513
rs7210574	0.247	0.916	0.810	0.238	0.646
rs3782735	0.876	0.540	0.471	0.241	0.021
rs1037169	0.261	0.891	0.726	0.245	0.381
rs10871794	0.230	0.252	0.131	0.246	0.466
rs7438888	0.790	0.682	0.171	0.250	0.364
4:128987952_ATT_A	0.349	0.199	0.131	0.251	0.587
rs35654957	0.756	0.588	0.817	0.251	0.168
rs186915841	0.077	0.730	0.997	0.253	0.313
rs71584764	0.668	0.610	0.165	0.255	0.014
rs62195072	0.616	0.648	0.884	0.259	0.112
rs144794875	0.157	0.061	0.301	0.264	0.056
rs116338429	0.823	0.244	0.110	0.264	0.160
rs773816354	0.274	0.380	0.358	0.265	0.222
rs3927496	0.984	0.506	0.334	0.267	0.126
11:65227616_CAAA_C	0.867	0.816	0.953	0.272	0.761
rs2197771	0.964	0.066	0.303	0.273	0.175
1:196914907_AGT_A	0.225	0.342	0.023	0.274	0.546
rs11994858	0.884	0.974	0.096	0.275	0.988
rs7808613	0.479	0.403	0.644	0.277	0.586
9:112204335_AT_A	0.357	0.172	0.730	0.278	0.116
rs11539938	0.435	0.389	0.003	0.280	0.557

rs1799831	0.222	0.487	0.820	0.282	0.026
rs6965401	0.729	0.702	0.451	0.284	0.923
rs757253787	0.141	0.031	0.968	0.291	0.082
12:93632423_CA_C	0.734	0.992	0.907	0.291	0.293
rs7655064	0.424	0.521	0.391	0.292	0.311
rs687339	0.415	0.879	0.128	0.294	0.238
rs55855238	0.637	0.573	0.842	0.298	0.027
rs11621792	0.514	0.033	0.361	0.302	0.754
rs72948115	0.346	0.829	0.649	0.303	0.840
rs73547906	0.270	0.029	0.832	0.304	0.112
rs1730865	0.395	0.654	0.073	0.305	0.997
rs4715316	0.162	0.905	0.528	0.306	0.101
2:111925731_CTTATGTT_C	0.939	0.786	0.513	0.307	0.429
9:123780856_AAAGGAAGG_A	0.279	0.762	0.693	0.309	0.689
rs8176526	0.814	0.693	0.912	0.313	0.005
rs501470	0.119	0.105	0.908	0.316	0.004
rs9379084	0.813	0.213	0.913	0.319	0.767
rs1871395	0.503	0.932	0.306	0.322	0.124
rs9266184	0.039	0.013	0.809	0.328	0.188
rs13108218	0.418	0.869	0.096	0.329	0.020
rs12696304	0.817	0.594	0.860	0.330	0.165
rs72708162	0.198	0.522	0.450	0.331	0.467
rs17185536	0.456	0.577	0.203	0.333	0.874
rs12818938	0.006	0.368	0.281	0.336	0.359
1:23747996_GA_G	0.715	0.996	0.760	0.338	0.235
rs904801	0.543	0.306	0.141	0.338	0.005
rs778239811	0.617	0.847	0.046	0.355	0.003
rs72683923	0.435	0.589	0.517	0.355	0.205
rs3006593	0.854	0.381	0.802	0.368	0.752
rs7623513	0.264	0.849	0.601	0.370	0.005
rs79717793	0.985	0.044	0.616	0.371	0.327
rs4077285	0.766	0.749	0.454	0.374	0.488
rs45490496	0.600	0.682	0.746	0.376	0.336
rs12797706	0.061	0.140	0.721	0.379	0.406
rs1640267	0.621	0.141	0.509	0.384	0.868
rs2905801	0.986	0.110	0.965	0.385	0.023
rs4645936	0.404	0.543	0.071	0.390	0.347
rs768159759	0.142	0.834	0.447	0.392	0.059
13:115013423_TTCTC_T	0.247	0.245	0.188	0.394	0.193
rs35225944	0.165	0.472	0.115	0.395	0.308
rs62442919	0.087	0.374	0.097	0.398	0.121
rs10083137	0.018	0.895	0.530	0.401	0.794
rs75349541	0.221	0.173	0.492	0.412	0.522

rs12690320	0.369	0.791	0.709	0.414	0.993
rs75170914	0.416	0.200	0.085	0.415	0.809
rs76895963	0.825	0.655	0.744	0.416	0.461
rs4333851	0.444	0.927	0.421	0.417	0.014
17:67080811_CT_C	0.342	0.800	0.057	0.418	0.948
rs56187480	0.472	0.422	0.124	0.419	0.160
rs8066941	0.858	0.686	0.405	0.421	0.955
X:133611871_AT_A	0.714	0.443	0.146	0.425	0.248
rs114165349	0.760	0.800	0.831	0.426	0.522
rs665731	0.074	0.159	0.843	0.426	0.453
1:1510035_GGC_G	0.058	0.161	0.570	0.427	0.533
rs2239222	0.664	0.742	0.857	0.429	0.413
rs10107182	0.919	0.717	0.115	0.430	0.934
rs2222018	0.401	0.812	0.343	0.433	0.025
rs41284816	0.834	0.203	0.058	0.433	0.988
rs111643014	0.985	0.427	0.496	0.433	0.608
rs8030357	0.222	0.819	0.830	0.437	0.430
rs211644	0.206	0.410	0.656	0.438	0.003
rs62394490	0.486	0.608	0.321	0.441	0.060
rs10411958	0.335	0.684	0.786	0.441	0.850
rs370654105	0.631	0.500	0.729	0.446	0.421
rs7694379	0.245	0.241	0.058	0.448	0.047
rs6900473	0.262	0.978	0.675	0.449	0.050
rs3747647	0.026	0.764	0.845	0.450	0.193
rs138529890	0.245	0.516	0.891	0.451	0.409
rs771435780	0.760	0.692	0.746	0.456	0.056
rs142627977	0.230	0.025	0.536	0.461	0.063
rs4979372	0.971	0.831	0.168	0.461	0.017
rs12059956	0.339	0.383	0.484	0.462	0.432
rs876435	0.749	0.347	0.232	0.468	0.189
rs11739158	0.920	0.521	0.109	0.469	0.549
rs1570516	0.137	0.978	0.449	0.479	0.358
rs2216707	0.088	0.078	0.172	0.480	0.725
rs13379043	0.489	0.250	0.959	0.481	0.438
rs56332871	0.695	0.538	0.278	0.489	0.245
rs11867902	0.334	0.605	0.090	0.491	0.331
rs2259305	0.360	0.392	0.411	0.496	0.371
rs1005421	0.105	0.347	0.413	0.497	0.265
rs10895277	0.074	0.010	0.849	0.500	0.195
rs820503	0.704	0.662	0.514	0.503	0.831
rs540730	0.078	0.018	0.752	0.512	0.105
rs202200760	0.447	0.949	0.845	0.514	0.942
5:36206855_GAT_G	0.296	0.140	0.577	0.515	0.659
rs71115649	0.152	0.713	0.242	0.515	0.493

rs2721195	0.822	0.641	0.759	0.519	0.615
rs2431752	0.938	0.365	0.116	0.521	0.932
rs1229492	0.737	0.744	0.948	0.530	0.847
9:19084633_CT_C	0.804	0.495	0.390	0.535	0.312
rs2234694	0.401	0.182	0.221	0.543	0.060
rs116483731	0.730	0.100	0.759	0.545	0.309
rs79354983	0.202	0.690	0.029	0.547	0.960
rs148911629	0.077	0.316	0.551	0.556	0.840
rs6736913	0.327	0.840	0.780	0.564	0.943
rs10153800	0.758	0.162	0.585	0.565	0.861
rs10657979	0.773	0.769	0.222	0.570	0.926
rs11641834	0.789	0.469	0.177	0.576	0.082
rs114949263	0.492	0.272	0.297	0.579	0.616
rs528350911	0.598	0.032	0.881	0.581	0.587
rs78444298	0.034	0.014	0.334	0.582	0.812
rs10116426	0.577	0.345	0.437	0.583	0.945
rs12454712	0.245	0.906	0.128	0.583	0.003
rs61929307	0.706	0.340	0.497	0.586	0.076
rs4782568	0.583	0.959	0.947	0.588	0.406
rs144647926	0.498	0.923	0.013	0.612	0.939
rs202128511	0.833	0.856	0.411	0.614	0.301
rs771193934	0.390	0.205	0.458	0.619	0.344
rs191884522	0.561	0.241	0.163	0.620	0.012
rs1782652	0.754	0.685	0.005	0.626	0.877
rs1351394	0.053	0.132	0.130	0.628	0.032
rs11601507	0.276	0.696	0.140	0.632	0.294
rs184640919	0.867	0.837	0.280	0.636	0.873
rs79287178	0.749	0.580	0.988	0.637	0.458
2:191559843_GT_G	0.387	0.456	0.964	0.640	0.528
8:81457499_CA_C	0.766	0.572	0.409	0.645	0.997
rs4860987	0.138	0.311	0.835	0.654	0.423
rs2820441	0.884	0.233	0.881	0.654	0.367
rs11666245	0.073	0.858	0.616	0.654	0.622
rs769447487	0.023	0.811	0.451	0.664	0.961
rs36103835	0.115	0.544	0.564	0.666	0.306
rs62134282	0.384	0.591	0.256	0.671	0.453
rs7314285	0.318	0.258	0.560	0.673	0.381
rs41412647	0.401	0.525	0.514	0.679	0.066
rs3812275	0.161	0.344	0.360	0.681	0.422
rs1991401	0.307	0.797	0.564	0.684	0.931
rs56196860	0.325	0.230	0.987	0.684	0.143
rs6480299	0.380	0.272	0.364	0.700	0.825
rs17583875	0.053	0.263	0.619	0.700	0.141
1:35734986_AAGTGCATCTTT_A	0.121	0.991	0.459	0.705	0.232

rs2247213	0.169	0.335	0.634	0.711	0.982
rs149663666	0.830	0.278	0.484	0.715	0.710
rs1890426	0.091	0.499	0.166	0.717	0.604
rs762450647	0.204	0.538	0.934	0.727	0.946
rs768651403	0.810	0.907	0.271	0.728	0.536
rs28650012	0.293	0.106	0.649	0.733	0.015
rs7096937	0.752	0.343	0.335	0.737	0.645
rs12543287	0.566	0.458	0.699	0.741	0.932
rs11655704	0.765	0.202	0.426	0.744	0.046
rs6792725	0.466	0.410	0.218	0.750	0.460
rs1870927	0.064	0.181	0.238	0.753	0.494
rs112352679	0.645	0.037	0.988	0.753	0.541
rs40270	0.733	0.717	0.139	0.753	0.986
rs7175361	0.296	0.920	0.289	0.754	0.841
rs198384	0.668	0.492	0.968	0.754	0.348
rs35234337	0.670	0.602	0.732	0.755	0.182
rs856534	0.897	0.531	0.563	0.756	0.232
rs75130744	0.617	0.152	0.857	0.757	0.612
rs56237852	0.268	0.373	0.145	0.759	0.624
rs34771269	0.525	0.968	0.855	0.767	0.258
rs8038465	0.124	0.576	0.949	0.769	0.253
rs111767734	0.086	0.079	0.701	0.771	0.987
rs60018147	0.250	0.863	0.486	0.773	0.455
rs9697210	0.065	0.375	0.859	0.775	0.835
rs71586027	0.002	0.069	0.261	0.784	0.155
rs180956060	0.216	0.253	0.772	0.789	0.355
rs780309846	0.249	0.543	0.386	0.790	0.170
rs29681	0.544	0.237	0.966	0.817	0.967
rs591939	0.164	0.179	0.445	0.831	0.000
rs73909848	0.706	0.778	0.656	0.832	0.240
rs246192	0.477	0.101	0.995	0.834	0.423
rs3780190	0.618	0.920	0.807	0.837	0.203
rs139974673	0.259	0.846	0.508	0.839	0.275
rs1788641	0.043	0.393	0.070	0.840	0.364
rs11732763	0.394	0.666	0.022	0.846	0.887
rs12928099	0.374	0.251	0.324	0.850	0.940
rs2564923	0.294	0.172	0.051	0.852	0.740
rs157935	0.780	0.508	0.044	0.861	0.029
rs12705095	0.992	0.861	0.039	0.865	0.341
rs1567353	0.108	0.092	0.750	0.870	0.440
rs13042148	0.614	0.112	0.116	0.873	0.787
rs565728741	0.200	0.235	0.772	0.877	0.660
rs775181992	0.428	0.335	0.339	0.879	0.300
rs78319058	0.238	0.744	0.969	0.883	0.180

rs7780562	0.002	0.161	0.171	0.884	0.127
rs148427769	0.184	0.963	0.019	0.885	0.795
rs72729610	0.869	0.782	0.180	0.889	0.168
rs4812336	0.842	0.826	0.029	0.893	0.705
rs201764793	0.814	0.503	0.341	0.895	0.459
rs6258	0.143	0.791	0.626	0.897	0.157
rs35824797	0.566	0.073	0.861	0.898	0.411
rs11856926	0.265	0.814	0.692	0.900	0.480
rs36086195	0.093	0.504	0.348	0.917	0.167
rs72663907	0.393	0.294	0.258	0.919	0.705
rs60005573	0.386	0.303	0.459	0.920	0.347
rs753645751	0.226	0.629	0.558	0.920	0.236
rs1349852	0.182	0.696	0.638	0.921	0.214
rs143709973	0.528	0.433	0.916	0.929	0.720
rs6595447	0.905	0.240	0.809	0.932	0.018
rs6939861	0.311	0.084	0.782	0.935	0.030
rs2156805	0.495	0.810	0.663	0.940	0.374
rs13389219	0.878	0.953	0.573	0.943	0.002
rs17747324	0.033	0.012	0.980	0.946	0.715
rs7735249	0.236	0.898	0.933	0.946	0.213
rs997907	0.186	0.889	0.019	0.953	0.077
rs11398692	0.494	0.109	0.655	0.953	0.379
rs9738226	0.518	0.114	0.086	0.953	0.313
rs2106854	0.982	0.372	0.921	0.953	0.542
rs35067979	0.786	0.913	0.613	0.957	0.271
rs174528	0.031	0.001	0.111	0.964	0.106
rs73631501	0.355	0.874	0.077	0.968	0.873
16:1786202_TGTGTGACCATCC_T	0.631	0.764	0.150	0.969	0.064
rs187325356	0.468	0.659	0.532	0.969	0.213
rs754959778	0.234	0.121	0.220	0.969	0.126
1:25827633_CT_C	0.313	0.024	0.971	0.978	0.619
rs2238799	0.035	0.065	0.017	0.983	0.208
rs1260326	0.150	0.187	0.352	0.985	0.000
rs7892835	0.117	0.327	0.560	0.986	0.153
rs2106727	0.590	0.053	0.292	0.987	0.909
rs10649697	0.967	0.580	0.498	0.988	0.333
rs17669311	0.562	0.546	0.972	0.989	0.127
rs28562483	0.266	0.657	0.781	0.989	0.314
rs61755050	0.158	0.674	0.744	0.990	0.083
rs1799941	0.850	0.199	0.319	0.993	0.148
3:49562992_CT_C	0.418	0.863	0.805	1.000	0.000

Supplementary Table 5. Associations of genetic predictors for SHBG with potential confounders in women

SNP	P value with potential confounders				
	Moderate physical activity	Vigorous physical activity	Townsend index	smoking	Alcohol drinking
1:155867257_CAA_C	0.036	0.453	0.959	0.791	0.040
1:221048577_TGA_T	0.576	0.989	0.931	0.689	0.193
1:23747996_GA_G	0.901	0.850	0.985	0.235	0.993
1:57170378_AATC_A	0.827	0.936	0.209	0.751	0.213
1:93787087_TA_T	0.876	0.661	0.228	0.317	0.969
10:65158772_AAAG_A	0.437	0.842	0.032	0.024	0.146
12:103483327_AT_A	0.281	0.329	0.026	0.024	0.773
12:12562340_GT_G	0.731	0.562	0.034	0.644	0.776
12:26614614_CAT_C	0.502	0.138	0.777	0.801	0.262
13:50565104_ACT_A	0.757	0.693	0.785	0.993	0.842
15:51052455_CT_C	0.157	0.807	0.959	0.731	0.266
15:53094375_TTTTG_T	0.893	0.154	0.035	0.194	0.875
17:48627860_CCT_C	0.485	0.648	0.590	0.338	0.206
19:7223973_TTTG_T	0.410	0.306	0.232	0.127	0.403
2:191559843_GT_G	0.287	0.800	0.785	0.348	0.875
2:208469213_ATCTT_A	0.851	0.240	0.129	0.097	0.835
3:122361173_TTTTTC_T	0.128	0.668	0.470	0.823	0.722
4:103877471_TA_T	0.014	0.601	0.564	0.058	0.019
6:130386212_GGAGA_G	0.246	0.035	0.094	0.758	0.462
7:130438531_CTTTTTT_C	0.005	0.551	0.913	0.857	0.110
8:59415339_TTG_T	0.775	0.124	0.569	0.344	0.345
8:81457499_CA_C	0.020	0.123	0.837	0.412	0.582
9:127474886_CA_C	0.994	0.191	0.133	0.422	0.004
rs1005421	0.717	0.433	0.844	0.982	0.357
rs10095380	0.826	0.701	0.769	0.720	0.198
rs10095930	0.314	0.460	0.292	0.548	0.958
rs10108150	0.184	0.344	0.760	0.536	0.410
rs10153315	0.160	0.809	0.896	0.451	0.891
rs10189479	0.836	0.980	0.098	0.366	0.201
rs10238028	0.646	0.076	0.697	0.284	0.276
rs1033667	0.142	0.113	0.034	0.150	0.832
rs1037169	0.708	0.560	0.824	0.232	0.803
rs10461018	0.586	0.477	0.954	0.367	0.852
rs1047891	0.541	0.033	0.559	0.697	0.812
rs10486782	0.354	0.561	0.705	0.693	0.065
rs10489206	0.165	0.363	0.689	0.743	0.743
rs10511002	0.076	0.133	0.974	0.355	0.051
rs10622246	0.797	0.581	0.281	0.657	0.811

rs10747689	0.072	0.032	0.678	0.014	0.172
rs10774095	0.041	0.388	0.118	0.659	0.461
rs10815276	0.317	0.704	0.725	0.619	0.540
rs10857228	0.058	0.413	0.380	0.373	0.484
rs10880868	0.403	0.504	0.400	0.222	0.153
rs10883451	0.083	0.045	0.628	0.658	0.020
rs10893876	0.598	0.873	0.472	0.416	0.189
rs10939934	0.375	0.845	0.784	0.266	0.412
rs10961205	0.756	0.390	0.045	0.332	0.111
rs11021232	0.745	0.333	0.037	0.502	0.332
rs11047237	0.790	0.828	0.793	0.538	0.163
rs11054861	0.727	0.312	0.888	0.076	0.127
rs11078597	0.555	0.452	0.667	0.501	0.955
rs11079685	0.313	0.701	0.650	0.711	0.504
rs11103377	0.656	0.397	0.761	0.178	0.016
rs111270317	0.090	0.015	0.773	0.015	0.985
rs111288118	0.325	0.945	0.027	0.903	0.521
rs11130982	0.246	0.404	0.161	0.899	0.191
rs11186719	0.640	0.804	0.558	0.537	0.383
rs11188601	0.244	0.897	0.830	0.201	0.560
rs112332688	0.871	0.745	0.259	0.092	0.816
rs1126161	0.626	0.553	0.113	0.190	0.686
rs1128249	0.087	0.615	0.761	0.095	0.001
rs112966033	0.183	0.773	0.373	0.726	0.024
rs113408476	0.556	0.861	0.355	0.824	0.288
rs11376788	0.545	0.191	0.685	0.773	0.601
rs114165349	0.630	0.789	0.466	0.309	0.694
rs114816312	0.199	0.494	0.260	0.658	0.872
rs114949263	0.570	0.309	0.729	0.406	0.570
rs115209326	0.646	0.354	0.842	0.317	0.344
rs11539938	0.556	0.772	0.963	0.469	0.744
rs11556924	0.930	0.499	0.284	0.044	0.528
rs11601507	0.523	0.146	0.714	0.153	0.504
rs11621792	0.616	0.236	0.842	0.159	0.351
rs116279971	0.083	0.341	0.852	0.271	0.031
rs11637595	0.938	0.661	0.666	0.418	0.650
rs11641834	0.139	0.434	0.925	0.022	0.409
rs11664106	0.815	0.613	0.622	0.263	0.036
rs11666245	0.024	0.110	0.082	0.322	0.955
rs11668201	0.681	0.925	0.730	0.038	0.017
rs11688682	0.489	0.457	0.891	0.006	0.440
rs11690748	0.522	0.782	0.508	0.295	0.010

rs11720108	0.586	0.117	0.030	0.737	0.045
rs11738093	0.949	0.408	0.498	0.504	0.075
rs117522510	0.246	0.898	0.477	0.301	0.062
rs11774700	0.828	0.996	0.290	0.282	0.035
rs11780978	0.649	0.159	0.868	0.687	0.609
rs11791747	0.460	0.311	0.089	0.714	0.017
rs11830764	0.683	0.815	0.703	0.161	0.243
rs11967262	0.584	0.857	0.996	0.412	0.359
rs12012896	0.031	0.064	0.002	0.204	0.100
rs12138803	0.472	0.577	0.159	0.457	0.335
rs1223796	0.631	0.447	0.044	0.922	0.143
rs12280075	0.547	0.526	0.020	0.263	0.050
rs1229498	0.699	0.254	0.321	0.456	0.528
rs12311848	0.891	0.745	0.905	0.070	0.479
rs12438742	0.830	0.761	0.369	0.740	0.964
rs12454712	0.276	0.295	0.456	0.217	0.312
rs12543287	0.331	0.528	0.065	0.208	0.957
rs12593818	0.199	0.029	0.692	0.205	0.387
rs1260326	0.541	0.025	0.740	0.022	0.000
rs12613243	0.286	0.624	0.819	0.193	0.773
rs12624244	0.231	0.105	0.690	0.840	0.738
rs12787996	0.241	0.866	0.277	0.791	0.165
rs12797706	0.870	0.266	0.349	0.151	0.381
rs12804411	0.920	0.195	0.949	0.606	0.845
rs12864658	0.924	0.100	0.715	0.489	0.864
rs12906447	0.105	0.543	0.940	0.622	0.891
rs13018007	0.858	0.597	0.841	0.118	0.008
rs13042148	0.078	0.940	0.905	0.091	0.379
rs13108218	0.927	0.155	0.396	0.027	0.000
rs13150068	0.082	0.753	0.855	0.242	0.868
rs13200245	0.523	0.449	0.129	0.391	0.481
rs13237750	0.635	0.212	0.480	0.064	0.952
rs1330307	0.581	0.631	0.453	0.919	0.241
rs13303359	0.293	0.373	0.369	0.165	0.199
rs13379043	0.066	0.604	0.325	0.152	0.025
rs13394092	0.424	0.303	0.312	0.201	0.615
rs138204164	0.623	0.731	0.140	0.652	0.573
rs139974673	0.072	0.186	0.181	0.157	0.258
rs140302625	0.925	0.978	0.174	0.492	0.099
rs140312320	0.431	0.634	0.185	0.643	0.985
rs140584594	0.730	0.306	0.681	0.426	0.197
rs1418652	0.132	0.690	0.709	0.680	0.111

rs1433210	0.664	0.042	0.906	0.110	0.001
rs143378550	0.363	0.448	0.744	0.563	0.338
rs146104952	0.830	0.306	0.932	0.382	0.039
rs146104952	0.902	0.214	0.973	0.427	0.058
rs147153202	0.683	0.325	0.874	0.982	0.751
rs148911629	0.860	0.784	0.043	0.878	0.494
rs150115323	0.515	0.628	0.602	0.248	0.323
rs150584036	0.017	0.096	0.028	0.961	0.034
rs1530439	0.965	0.629	0.927	0.161	0.275
rs1634791	0.109	0.988	0.480	0.329	0.365
rs1650527	0.190	0.775	0.159	0.863	0.315
rs1684608	0.862	0.061	0.118	0.534	0.418
rs16995626	0.264	0.095	0.371	0.374	0.455
rs17128091	0.400	0.902	0.183	0.103	0.609
rs17202341	0.430	0.165	0.819	0.698	0.703
rs1730862	0.453	0.679	0.413	0.116	0.981
rs1738386	0.920	0.272	0.446	0.390	0.630
rs1741344	0.259	0.020	0.605	0.426	0.356
rs174537	0.687	0.550	0.008	0.622	0.621
rs17492269	0.787	0.095	0.390	0.341	0.751
rs17580	0.920	0.240	0.962	0.937	0.069
rs17583875	0.625	0.347	0.649	0.156	0.965
rs17616365	0.035	0.462	0.040	0.483	0.441
rs17669311	0.289	0.718	0.901	0.699	0.146
rs1782652	0.416	0.966	0.241	0.638	0.776
rs17887160	0.331	0.110	0.146	0.807	0.327
rs1801282	0.289	0.855	0.815	0.444	0.654
rs183015141	0.620	0.857	0.911	0.329	0.733
rs185044544	0.420	0.094	0.949	0.012	0.789
rs1870927	0.218	0.825	0.135	0.906	0.166
rs1872992	0.300	0.230	0.231	0.033	0.562
rs1962883	0.113	0.100	0.115	0.413	0.264
rs198358	0.545	0.954	0.064	0.900	0.092
rs199607859	0.288	0.036	0.600	0.592	0.689
rs2009310	0.035	0.034	0.835	0.761	0.032
rs201468966	0.195	0.224	0.017	0.117	0.045
rs2018519	0.575	0.999	0.305	0.517	0.068
rs201874554	0.545	0.851	0.643	0.295	0.265
rs202021413	0.001	0.001	0.372	0.783	0.481
rs202200760	0.128	0.402	0.887	0.670	0.464
rs2057655	0.737	0.949	0.124	0.960	0.800
rs2064074	0.670	0.945	0.879	0.852	0.980

rs2068888	0.063	0.855	0.948	0.459	0.557
rs2176040	0.230	0.520	0.595	0.101	0.008
rs2207132	0.144	0.067	0.226	0.446	0.658
rs2239222	0.398	0.734	0.689	0.975	0.106
rs2246223	0.002	0.608	0.016	0.072	0.151
rs2288926	0.972	0.933	0.697	0.170	0.845
rs2299055	0.247	0.263	0.834	0.999	0.411
rs2364717	0.400	0.040	0.467	0.786	0.191
rs2438109	0.392	0.530	0.392	0.398	0.498
rs2498786	0.101	0.702	0.886	0.574	0.657
rs2525570	0.945	0.916	0.336	0.896	0.000
rs267733	0.752	0.257	0.350	0.003	0.470
rs2723555	0.310	0.147	0.862	0.350	0.136
rs275177	0.726	0.370	0.744	0.225	0.493
rs28360642	0.749	0.099	0.762	0.012	0.224
rs28459049	0.374	0.238	0.595	0.952	0.625
rs28636815	0.270	0.648	0.736	0.876	0.115
rs28712547	0.620	0.806	0.789	0.678	0.911
rs28925904	0.445	0.850	0.054	0.124	0.022
rs28929470	0.749	0.669	0.371	0.177	0.318
rs28929474	0.293	0.043	0.504	0.004	0.029
rs2915023	0.134	0.266	0.834	0.925	0.621
rs2924808	0.065	0.539	0.644	0.928	0.164
rs2925979	0.354	0.120	0.951	0.888	0.732
rs2970871	0.522	0.062	0.108	0.594	0.400
rs2980858	0.187	0.728	0.135	0.414	0.008
rs2986669	0.741	0.231	0.927	0.478	0.998
rs3001032	0.102	0.638	0.074	0.461	0.334
rs3018695	0.092	0.925	0.193	0.333	0.601
rs34154818	0.282	0.074	0.887	0.749	0.309
rs34184867	0.530	0.438	0.222	0.750	0.208
rs34255979	0.781	0.454	0.448	0.045	0.988
rs34311866	0.933	0.834	0.996	0.006	0.819
rs34331968	0.173	0.842	0.063	0.702	0.649
rs34385891	0.844	0.802	0.083	0.676	0.529
rs34499031	0.638	0.712	0.456	0.339	0.105
rs34651	0.559	0.448	0.970	0.894	0.788
rs35143646	0.552	0.880	0.150	0.585	0.157
rs35198068	0.520	0.565	0.119	0.408	0.565
rs35233014	0.779	0.565	0.598	0.212	0.110
rs35475471	0.147	0.034	0.249	0.542	0.737
rs35568851	0.622	0.158	0.242	0.734	0.725

rs35696875	0.159	0.316	0.563	0.550	0.591
rs3733321	0.046	0.592	0.123	0.803	0.973
rs3747207	0.857	0.451	0.093	0.333	0.088
rs3747367	0.355	0.012	0.650	0.973	0.982
rs3749228	0.150	0.794	0.204	0.777	0.002
rs3751129	0.432	0.930	0.035	0.000	0.120
rs3768420	0.742	0.611	0.596	0.748	0.860
rs3782735	0.743	0.634	0.185	0.317	0.916
rs390408	0.261	0.019	0.731	0.803	0.332
rs40270	0.105	0.698	0.891	0.368	0.108
rs4077285	0.029	0.995	0.565	0.950	0.383
rs4092465	0.082	0.661	0.305	0.997	0.002
rs4122352	0.187	0.561	0.488	0.042	0.900
rs41280463	0.385	0.992	0.648	0.649	0.105
rs4149056	0.014	0.260	0.528	0.895	0.204
rs4264433	0.721	0.016	0.589	0.828	0.014
rs4307773	0.780	0.312	0.279	0.243	0.908
rs4327143	0.661	0.393	0.179	0.625	0.292
rs4450871	0.456	0.724	0.995	0.503	0.901
rs4530527	0.105	0.612	0.014	0.175	0.318
rs4563785	0.510	0.849	0.598	0.130	0.333
rs469721	0.316	0.252	0.516	0.762	0.024
rs4709746	0.046	0.381	0.911	0.925	0.985
rs4804669	0.680	0.263	0.118	0.161	0.447
rs4810580	0.789	0.944	0.084	0.329	0.120
rs4830411	0.537	0.367	0.853	0.505	0.026
rs4837794	0.695	0.975	0.427	0.021	0.011
rs4871015	0.700	0.481	0.975	0.380	0.191
rs4876993	0.169	0.115	0.026	0.670	0.929
rs4976033	0.908	0.777	0.258	0.978	0.155
rs5112	0.286	0.510	0.666	0.789	0.901
rs5117	0.000	0.045	0.055	0.118	0.395
rs528350911	0.047	0.671	0.690	0.125	0.565
rs528806375	0.411	0.209	0.882	0.153	0.064
rs545206972	0.316	0.956	0.265	0.746	0.483
rs548235873	0.268	0.255	0.012	0.000	0.078
rs549664712	0.886	0.945	0.233	0.178	0.000
rs550767741	0.680	0.408	0.138	0.240	0.623
rs555234816	0.444	0.485	0.902	0.500	0.915
rs555754	0.165	0.612	0.358	0.922	0.010
rs56332871	0.247	0.287	0.114	0.239	0.295
rs568656	0.681	0.107	0.841	0.485	0.104

rs57158761	0.457	0.545	0.691	0.231	0.160
rs5750131	0.285	0.391	0.733	0.098	0.401
rs5753111	0.863	0.555	0.221	0.345	0.198
rs5791099	0.133	0.400	0.666	0.078	0.633
rs5813220	0.732	0.801	0.300	0.605	0.371
rs5820605	0.428	0.083	0.007	0.606	0.000
rs58321169	0.468	0.277	0.335	0.095	0.157
rs58489806	0.033	0.090	0.300	0.831	0.259
rs59774409	0.172	0.361	0.578	0.874	0.499
rs59837038	0.788	0.414	0.431	0.018	0.904
rs60018147	0.295	0.778	0.436	0.541	0.020
rs6058067	0.318	0.588	0.824	0.681	0.717
rs6073431	0.934	0.599	0.245	0.225	0.105
rs6088776	0.291	0.749	0.453	0.046	0.147
rs6129778	0.248	0.764	0.410	0.651	0.048
rs6129778	0.246	0.155	0.725	0.370	0.786
rs61755050	0.757	0.520	0.128	0.078	0.313
rs61830291	0.459	0.151	0.959	0.621	0.047
rs62128735	0.113	0.146	0.419	0.204	0.034
rs62186584	0.673	0.033	0.044	0.106	0.766
rs62271373	0.845	0.974	0.200	0.356	0.001
rs62303689	0.935	0.020	0.641	0.530	0.965
rs62486442	0.972	0.149	0.211	0.610	0.908
rs62515079	0.705	0.204	0.643	0.490	0.108
rs62580766	0.128	0.028	0.116	0.330	0.444
rs6531735	0.518	0.158	0.049	0.564	0.508
rs6546096	0.436	0.517	0.809	0.637	0.393
rs6706	0.804	0.098	0.707	0.424	0.312
rs6736913	0.539	0.884	0.977	0.864	0.966
rs67651018	0.289	0.474	0.357	0.218	0.240
rs6772177	0.534	0.053	0.650	0.851	0.180
rs67890964	0.863	0.653	0.272	0.086	0.795
rs6792725	0.032	0.391	0.406	0.875	0.103
rs68002561	0.500	0.291	0.323	0.794	0.218
rs6803518	0.153	0.485	0.287	0.296	0.685
rs6831257	0.071	0.548	0.255	0.346	0.386
rs6860245	0.810	0.946	0.964	0.771	0.059
rs687339	0.379	0.876	0.523	0.664	0.204
rs6879874	0.775	0.856	0.771	0.825	0.689
rs696825	0.321	0.238	0.660	0.295	0.919
rs7139079	0.787	0.561	0.329	0.024	0.180
rs71468663	0.862	0.527	0.412	0.477	0.604

rs71531849	0.294	0.498	0.687	0.188	0.443
rs7250869	0.505	0.712	0.746	0.006	0.828
rs7252372	0.778	0.048	0.429	0.345	0.671
rs72681869	0.463	0.915	0.093	0.065	0.041
rs72766607	0.338	0.649	0.525	0.674	0.032
rs72782727	0.341	0.484	0.218	0.069	0.213
rs72836346	0.695	0.902	0.003	0.146	0.130
rs72844546	0.338	0.468	0.852	0.017	0.571
rs7298820	0.544	0.962	0.975	0.866	0.434
rs73036519	0.754	0.426	0.051	0.504	0.005
rs7321688	0.015	0.340	0.694	0.703	0.427
rs73519353	0.777	0.932	0.946	0.989	0.329
rs73519353	0.142	0.042	0.345	0.470	0.917
rs73670309	0.474	0.157	0.214	0.221	0.023
rs740516	0.060	0.002	0.586	0.334	0.278
rs740893	0.291	0.625	0.775	0.383	0.368
rs74090351	0.164	0.376	0.867	0.188	0.492
rs7475279	0.624	0.786	0.967	0.507	0.686
rs7481219	0.972	0.843	0.841	0.837	0.718
rs7484541	0.122	0.614	0.448	0.945	0.760
rs75077113	0.010	0.094	0.486	0.476	0.655
rs75130744	0.321	0.936	0.774	0.771	1.000
rs7567544	0.783	0.706	0.417	0.149	0.498
rs764029425	0.693	0.060	0.333	0.331	0.150
rs76491020	0.975	0.301	0.602	0.683	0.525
rs76767219	0.819	0.683	0.009	0.421	0.004
rs768159759	0.134	0.070	0.982	0.179	0.593
rs76895963	0.686	0.395	0.827	0.597	0.127
rs7696472	0.010	0.019	0.207	0.586	0.931
rs770971500	0.667	0.597	0.853	0.091	0.187
rs771193934	0.015	0.840	0.021	0.836	0.900
rs775181992	0.451	0.557	0.661	0.569	0.961
rs778571122	0.943	0.339	0.453	0.575	0.004
rs78057960	0.241	0.617	0.465	0.908	0.632
rs781996653	0.595	0.055	0.340	0.513	0.008
rs784504	0.762	0.969	0.884	0.497	0.866
rs78890745	0.999	0.704	0.776	0.155	0.327
rs79237700	0.942	0.524	0.992	0.788	0.824
rs79287178	0.259	0.647	0.587	0.737	0.195
rs79391862	0.527	0.132	0.554	0.440	0.665
rs799157	0.147	0.750	0.941	0.883	0.045
rs80126506	0.963	0.768	0.429	0.893	0.280

rs80235628	0.750	0.453	0.066	0.946	0.119
rs8027064	0.500	0.515	0.863	0.533	0.890
rs8107967	0.383	0.292	0.163	0.547	0.417
rs8176741	0.392	0.596	0.159	0.368	0.129
rs8178824	0.062	0.517	0.408	0.187	0.167
rs820504	0.011	0.568	0.049	0.473	0.599
rs848476	0.296	0.183	0.196	0.795	0.544
rs858519	0.011	0.754	0.824	0.270	0.189
rs868655	0.111	0.412	0.253	0.897	0.208
rs8756	0.039	0.188	0.546	0.018	0.000
rs892225	0.263	0.773	0.145	0.686	0.365
rs899865	0.039	0.154	0.790	0.305	0.310
rs921153	0.088	0.799	0.626	0.033	0.503
rs925098	0.793	0.614	0.400	0.445	0.068
rs9366291	0.501	0.990	0.173	0.055	0.836
rs9379084	0.102	0.119	0.831	0.208	0.007
rs9426829	0.208	0.927	0.106	0.722	0.282
rs9461793	0.456	0.318	0.197	0.408	0.083
rs9556403	0.608	0.464	0.506	0.432	0.009
rs9644032	0.445	0.765	0.206	0.967	0.285
rs9697210	0.533	0.393	0.940	0.121	0.604
rs9834503	0.579	0.720	0.971	1.000	0.771
rs9872754	0.562	0.683	0.768	0.302	0.365
rs9987289	0.986	0.717	0.570	0.362	0.001

Supplementary Table 6. Sex-specific associations of genetic predictors for SHBG with LDL-cholesterol and lipoprotein (a)

Outcome	Sex	beta	95% CI	*p*	MR Egger intercept *p*
LDL-cholesterol	Men	-0.06	-0.15, 0.04	0.24	0.60
	Women	-0.05	-0.12, 0.03	0.23	0.87
Lipoprotein (a)	Men	-0.07	-0.17, 0.04	0.21	0.43
	Women	-0.02	-0.11, 0.07	0.67	0.88

Inverse variance weighting was used in men and women because the MR Egger intercept p value does not indicate pleiotropy (intercept p>0.05).

Supplementary Table S7. Sex-specific associations of genetically predicted SHBG with ischemic heart disease in the UK Biobank excluding genetic variants related to BMI in univariable MR

Outcome	Sex	OR	95% CI	*p*	MR Egger intercept *p*
IHD	Men	0.79	0.71, 0.89	4.3×10^{-5}	0.48
	Women	0.93	0.78, 1.10	0.41	0.04

IHD, ischemic heart disease. 4 SNPs related to BMI in men and 2 SNPs related to BMI in women were excluded. Inverse variance weighting was used in men and MR Egger corrected estimates were used in women because the MR Egger intercept p value<0.05.

Supplemental Table S8. Sex-specific associations of genetically predicted SHBG with ischemic heart disease in the UK Biobank using non-overlapping samples in the UK Biobank

Outcome	Sex	OR	95% CI	*p*	MR Egger intercept *p*
IHD	Men	0.79	0.71, 0.88	3.3×10^{-5}	0.52
	Women	0.93	0.78, 1.10	0.38	0.02

IHD, ischemic heart disease. Inverse variance weighting was used in men and MR Egger corrected estimates were used in women because the MR Egger intercept p value<0.05.

References

1. Ruth KS, Day FR, Tyrrell J, Thompson DJ, Wood AR, Mahajan A et al: Using human genetics to understand the disease impacts of testosterone in men and women. Nat Med 2020, 26(2):252-258.

Supplementary Figure S1. Flow chart of the study design

(a) Univariable Mendelian randomization

Europeans

Sex-specific genetic predictors for SHBG

↓

Genetic association with ischemic heart disease in men and women

Japanese

Genetic predictors for SHBG in men

↓

Genetic association with ischemic heart disease in men

(b) Multivariable Mendelian randomization controlling for bioavailable testosterone in men

Europeans

Genetic predictors for SHBG and bioavailable testosterone in men

↓

Genetic association with ischemic heart disease in men

Supplementary Figure S2. Scatter plot on the genetic association with sex hormone binding globulin and with ischemic heart disease in men and women in the UK Biobank

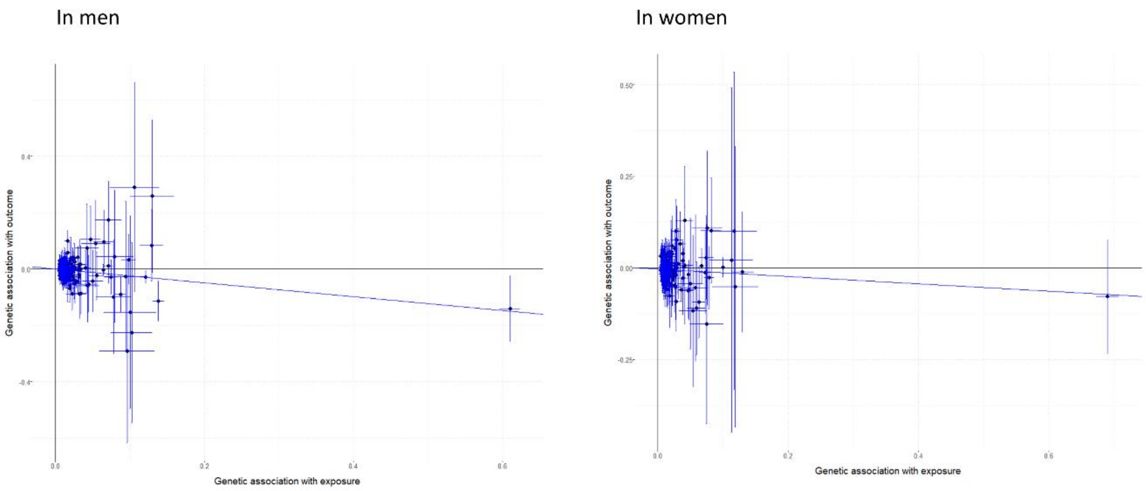

Supplementary Figure S3. Leave-one-out analysis on the sex-specific association of genetically predicted sex hormone binding globulin with ischemic heart disease in the UK Biobank

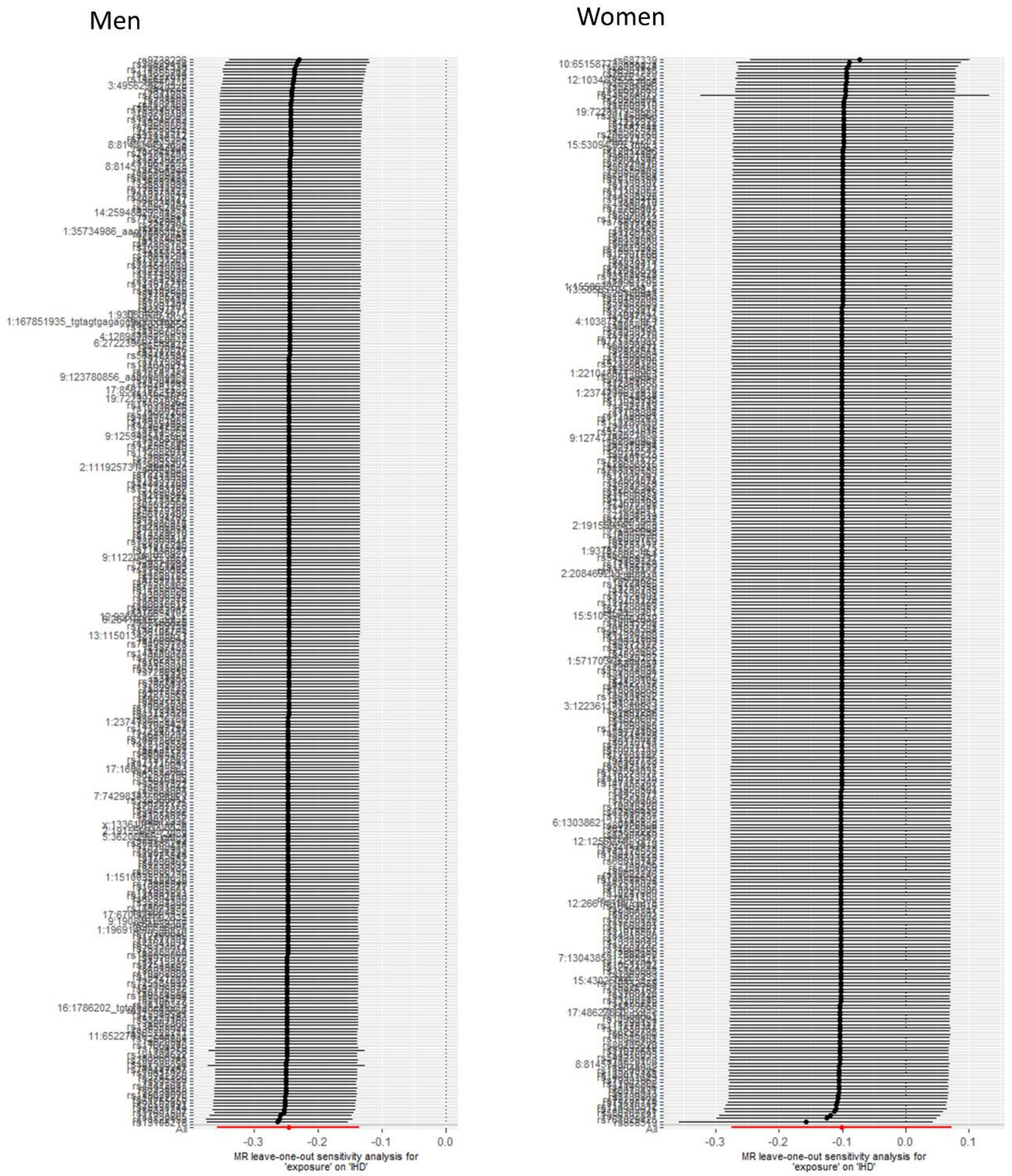

Supplement: Supplementary file 2 — Supplementary Information 2. [file 41598_2021_2510_MOESM2_ESM.pdf]
